# Global long-term observations of coastal erosion and accretion

**DOI:** 10.1038/s41598-018-30904-w

**Published:** 2018-08-27

**Authors:** Lorenzo Mentaschi, Michalis I. Vousdoukas, Jean-Francois Pekel, Evangelos Voukouvalas, Luc Feyen

**Affiliations:** 1 European Commission, Joint Research Centre (JRC), Directorate for Space Security and Migration, Via Enrico Fermi 2749, 21027, Ispra, Italy; 2 Department of Marine Sciences, University of the Aegean, Mitilene, Lesbos, Greece; 3 European Commission, Joint Research Centre (JRC), Directorate for Sustainable Resources, Via Enrico Fermi 2749, 21027, Ispra, Italy; 4 Engineering Ingegneria Informatica s.p.a., Via S. Martino della Battaglia, 56, 00185, Rome, Italy

## Abstract

Changes in coastal morphology have broad consequences for the sustainability of coastal communities, structures and ecosystems. Although coasts are monitored locally in many places, understanding long-term changes at a global scale remains a challenge. Here we present a global and consistent evaluation of coastal morphodynamics over 32 years (1984–2015) based on satellite observations. Land losses and gains were estimated from the changes in water presence along more than 2 million virtual transects. We find that the overall surface of eroded land is about 28,000 km^2^, twice the surface of gained land, and that often the extent of erosion and accretion is in the order of km. Anthropogenic factors clearly emerge as the dominant driver of change, both as planned exploitation of coastal resources, such as building coastal structures, and as unforeseen side effects of human activities, for example the installment of dams, irrigation systems and structures that modify the flux of sediments, or the clearing of coastal ecosystems, such as mangrove forests. Another important driver is the occurrence of natural disasters such as tsunamis and extreme storms. The observed global trend in coastal erosion could be enhanced by Sea Level Rise and more frequent extreme events under a changing climate.

## Introduction

Coastal environments form the interface between the land and sea or ocean. They host key infrastructures, ecosystems and about 40% of the world’s global population^1,2^. The buffer area between permanent land and water is occasionally submerged, due to the action of tides, waves and rivers in the estuarine zones. This area defines the coastal active zone (see the methods section), which is a highly dynamic area that provides protection from coastal natural hazards by absorbing energy and momentum fluxes from the ocean^3^, and that hosts a wide range of precious marine biosystems^4^.

Our coasts undergo constant changes as rivers, nearshore currents and waves move sediments inside, outside and within the nearshore zone^5,6^. Morphological evolution tends to accelerate under extreme events, such as tsunamis, storms, and tropical cyclones that drive intense erosion and can lead to irreversible changes^7–10^. Human presence also leaves a strong footprint, either through planned exploitation of coastal resources^11^ or as a side effect of activities that result in deterioration of the coastal environment^12–15^. Moreover, relative sea-level rise (RSLR) contributes to coastal erosion, especially in low-lying and flat areas, through a complex morphological adaptation, and by increasing the exposure to other drivers of morphodynamics^16,17^.

A number of techniques have been developed to monitor coastal morphology dynamics, such as direct measuring of distance and monitoring with laser, cameras or aerial photography^18–22^. Their application has resulted in a better understanding of coastal change patterns at local and regional scales. These studies are generally characterized by high resolution data for rather small areas. Their local character, together with the use of different measures of change (e.g. cross-shore lengths referred to different estimated coastlines, or surfaces, or surface per year of erosion/accretion) and differing space-time settings, hamper their use for comparative analyses. Furthermore, because these techniques are demanding in terms of manpower, equipment and costs, it is difficult to deploy them at larger scales. This makes remote sensing a very attractive option. Although in the last decade, studies have used space observations for detecting local coastal morphological change^8,23–25^, a comprehensive global analysis of shoreline evolution is still lacking, but is now made possible by the availability of big data facilities and frameworks dedicated to the storage and elaboration of satellite data^26,27^. Recently^28^ presented a global study, yet they focus exclusively on sand beaches.

Exploiting the extensive monitoring capabilities of satellites^29,30^, here we developed a global-scale and consistent database of coastal morphodynamics, valid for any type of shore. This study is based on the Global Surface Water Explorer (GSWE) dataset, a global database derived from the analysis of over 3 million satellite images that maps water presence over 32 years^29^. This dataset was analyzed for changes in water presence along more than 2 million virtual transects (see Methods section and SI). The spatial resolution of our analysis is 30 m in the cross-shore direction, corresponding to the resolution of the GSWE dataset (based on Landsat imagery, courtesy of the U.S. Geological Survey, USGS), and 250 m in the long-shore direction (i.e. the distance between transects).

The remainder of this paper is structured as follows: in Section 2 the results are summarized; in Section 3 the results are discussed, examining extreme and noticeable cases of erosion/accretion and relating these with known drivers of coastal morphodynamics; in Section 4 the conclusions drawn from the study are presented.

## Results

The result of our analysis consists of an estimation of lost and gained land for each coastal transect. Lost land identifies areas previously permanently dry that transited to wet or partially wet. Gained land coincides with surfaces that transited from wet or partially wet to permanently dry. The processing was performed thanks to the petascale computational power of Google Earth Engine^26^.

**Figure 1:**
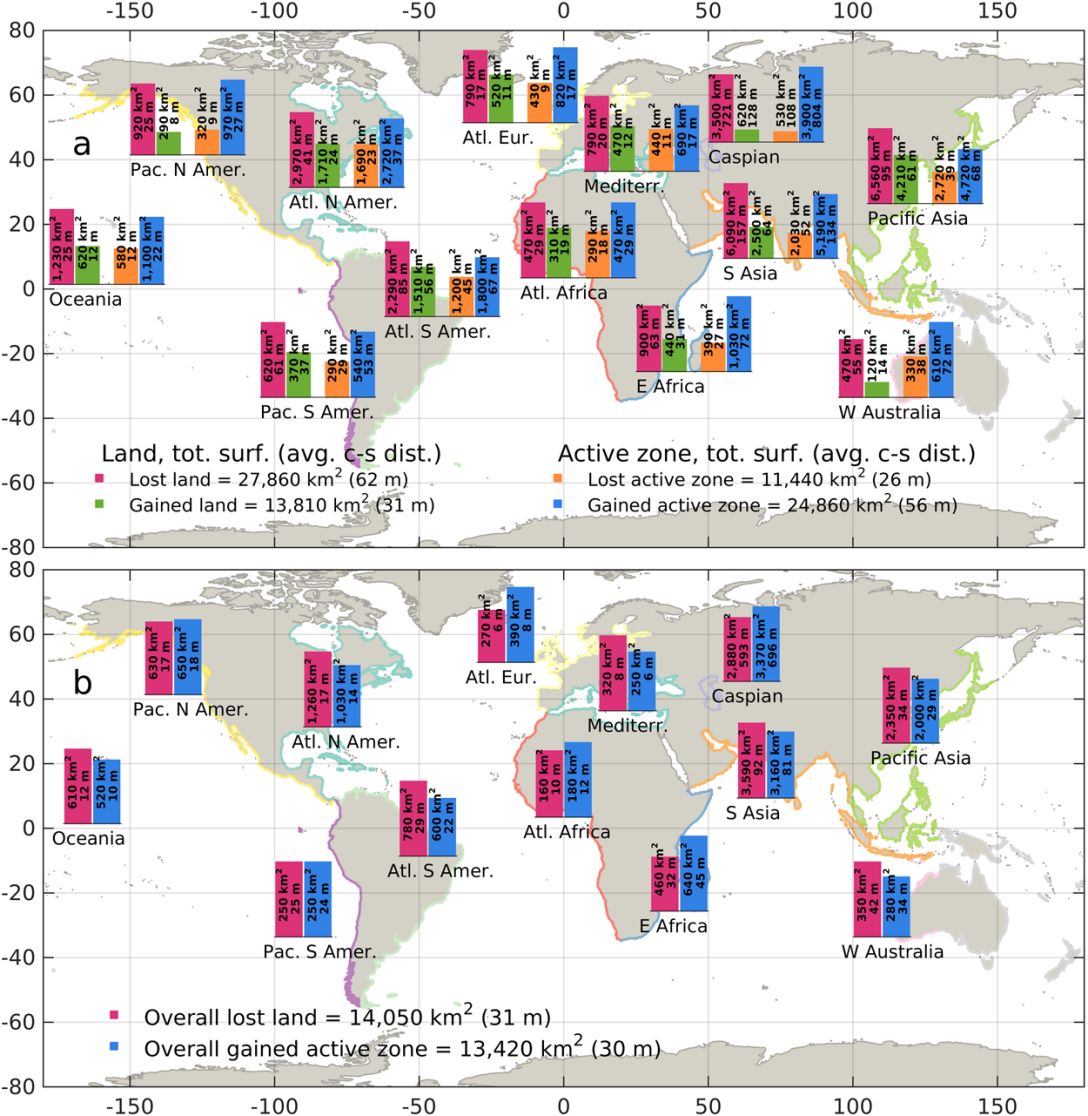
Overall gained and lost **(a)**, and gained-lost neat balance **(b)** of land and active zone, aggregated by continent/ocean and expressed in km^2^ and in cross-shore distance. The global aggregated quantities are also shown in both panels. Coastline colors identify the considered areas. This figure was generated with the MATLAB programming language.

On a global scale, between 1984 and 2015, the loss of permanent land in coastal areas amounts to almost 28,000 km^2^, roughly equivalent to the surface area of Haiti (Fig. 1a). This is almost twice as large as the surface of gained land (about 14,000 km^2^) over the same period. On the other hand, the overall surface of gained active zone (about 25,000 km^2^) is more than two times larger than the surface of lost active zone (about 11,500 km^2^). Overall, the gain of active zone roughly balances the loss of land, and the gain of land balances the loss of active zone. This translates into a net loss of approximately 14,000 km^2^ of surface for human settlements and terrestrial ecosystems (Fig. 1b).

Regionally aggregated results show that for each continent or ocean the amount of eroded surface area outweighs that of accreted land, and that the active zone expands mainly by encroaching into previously permanent land. The region with the highest change per unit coast is the Caspian Sea (about 600 m of average net cross-shore land loss and 700 m of active zone gain, Fig. 1a), followed by Southern Asia (with an average land erosion of 158 m partially balanced by an accretion of 69 m, Fig. 1a). Also, Pacific Asia, Southern America, Eastern Africa and Western Australia present an average cross-shore erosion above 50 m (Fig. 1a). More than 50% of the overall global changes take place along Asian and Caspian coasts.

**Figure 2:**
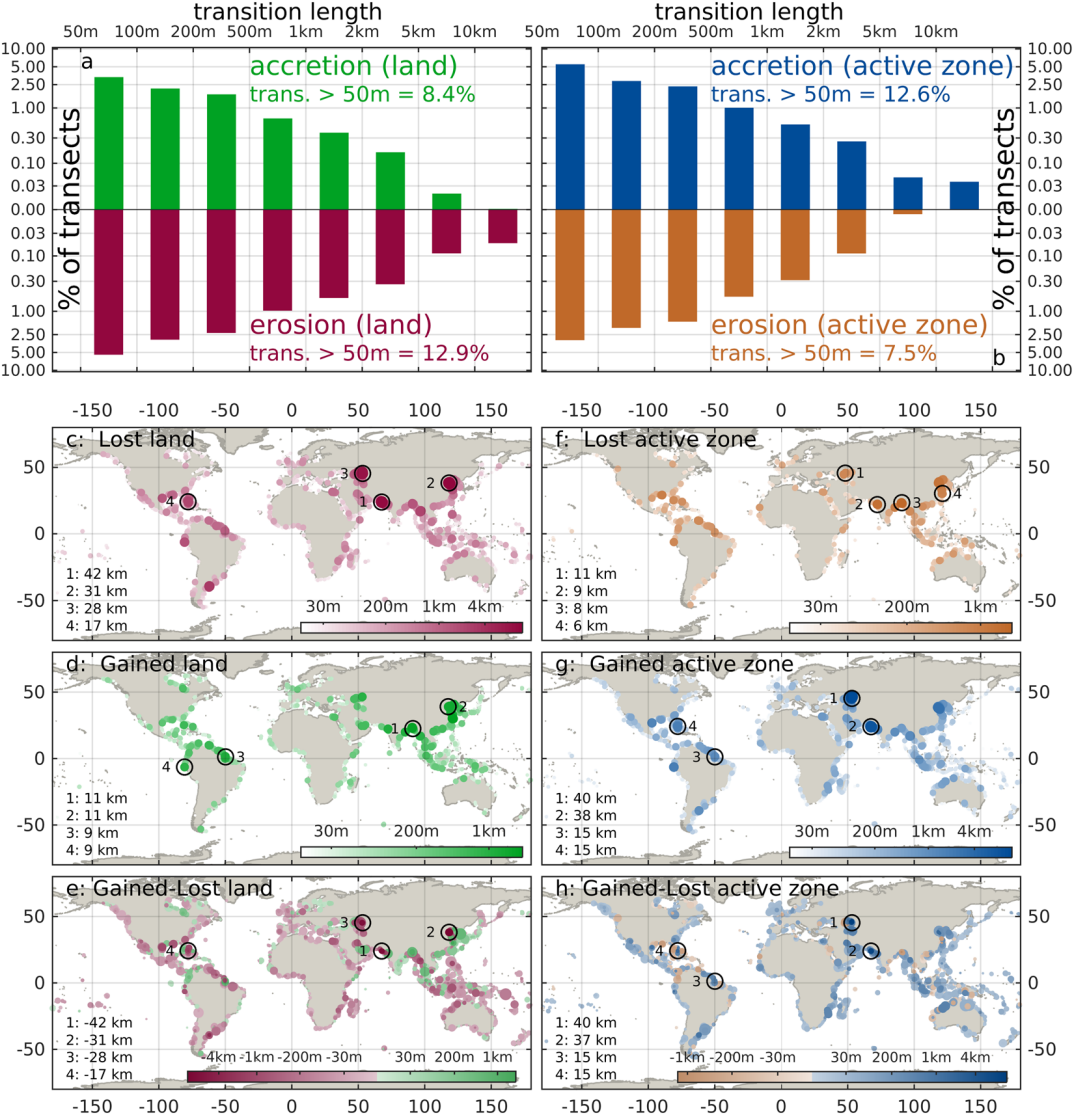
Size distribution of cross-shore transition length above 50 m, for erosion and accretion of land **(a)** and active zone **(b)**. Lost **(c)** and gained **(d)** land around the world; lost **(f)** and gained **(g)** active zone; balance gained - lost land **(e)** and active zone **(h)**. Maps show the length of cross-shore erosion and accretion aggregated on coastal segments of 100 km. In all the maps the 4 spots with the highest local transition along a 250 m transect are indicated. This figure was generated with the MATLAB programming language.

The size of the observed changes can differ strongly between coastal stretches. The length of cross-shore land erosion or active zone accretion exceeds 50 m in about 13% of the transects, while land accretion or active zone erosion exceeds the same threshold in about 8% of the transects (Fig. 2a,b). The transition length in single transects can exceed one km (in about 1.8% of the transects), and locally even reach tens of km (in about 0.07% of the transects). Outstanding examples of this are the Indus delta (Fig. 2c, location 1), some parts of the Bohai coasts (Fig. 2c,d, location 2) and along the Caspian Kazakh coast (Fig. 2c, location 3).

## Discussion

**Figure 3:**
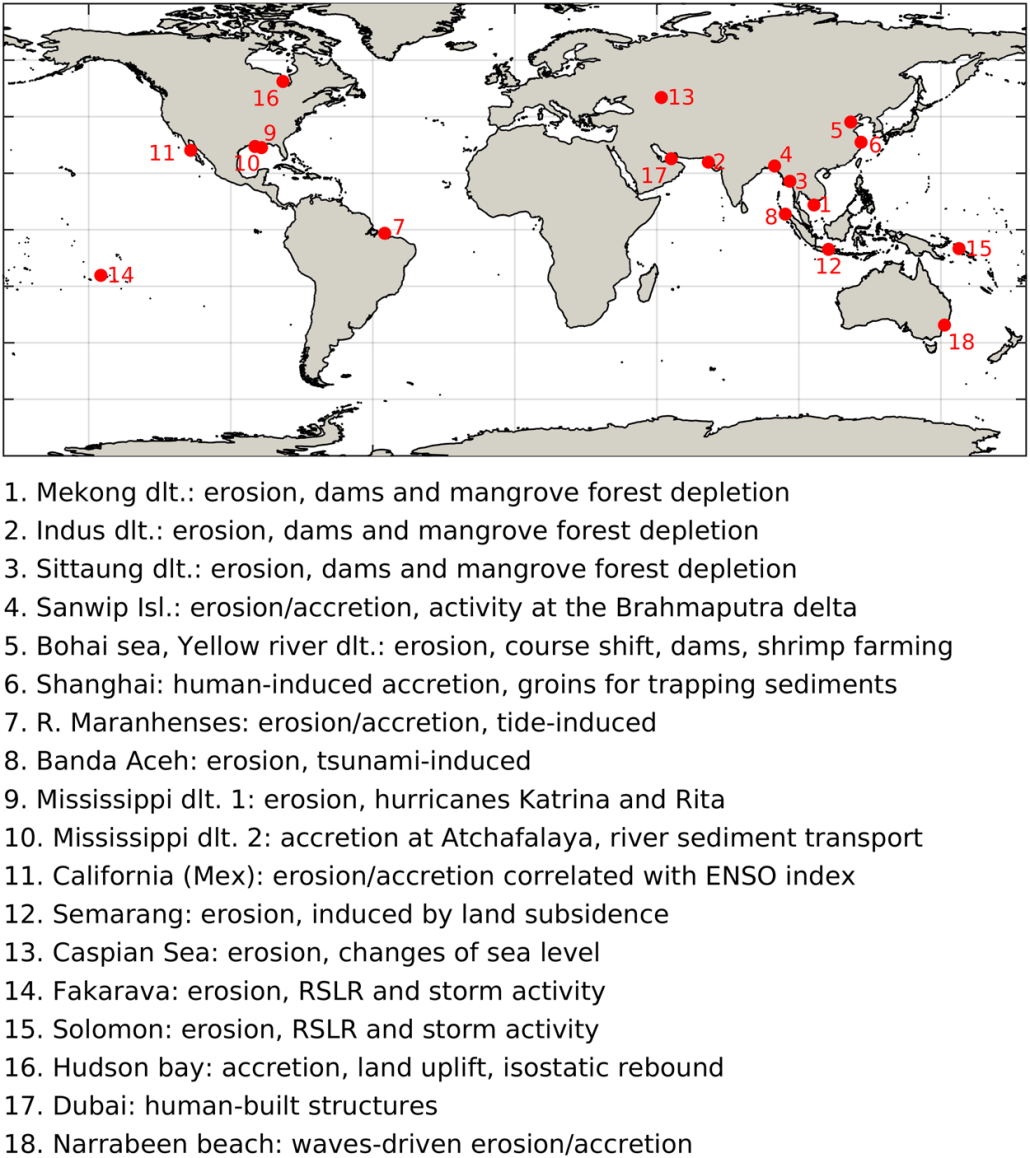
List of local cases of erosion/accretion discussed in this manuscript or used for the dataset validation. The legend provides for each spot a brief summary of the drivers of shoreline change.

In this section the results are discussed and explained in terms of the different drivers and trends that characterize local changes of shoreline. The examples illustrated here (Fig. 3) were selected in order to explain the most extreme observed cases of coastal erosion or accretion, to provide notable examples of the known major sources of coastal morphodynamics, and to verify the presence of documented tendencies. For many of the described cases, our estimates have been compared with those from existing local studies (see section 1 of SI), which also provided supporting arguments for the discussion of the drivers of change.

### Anthropogenic factors

**Figure 4:**
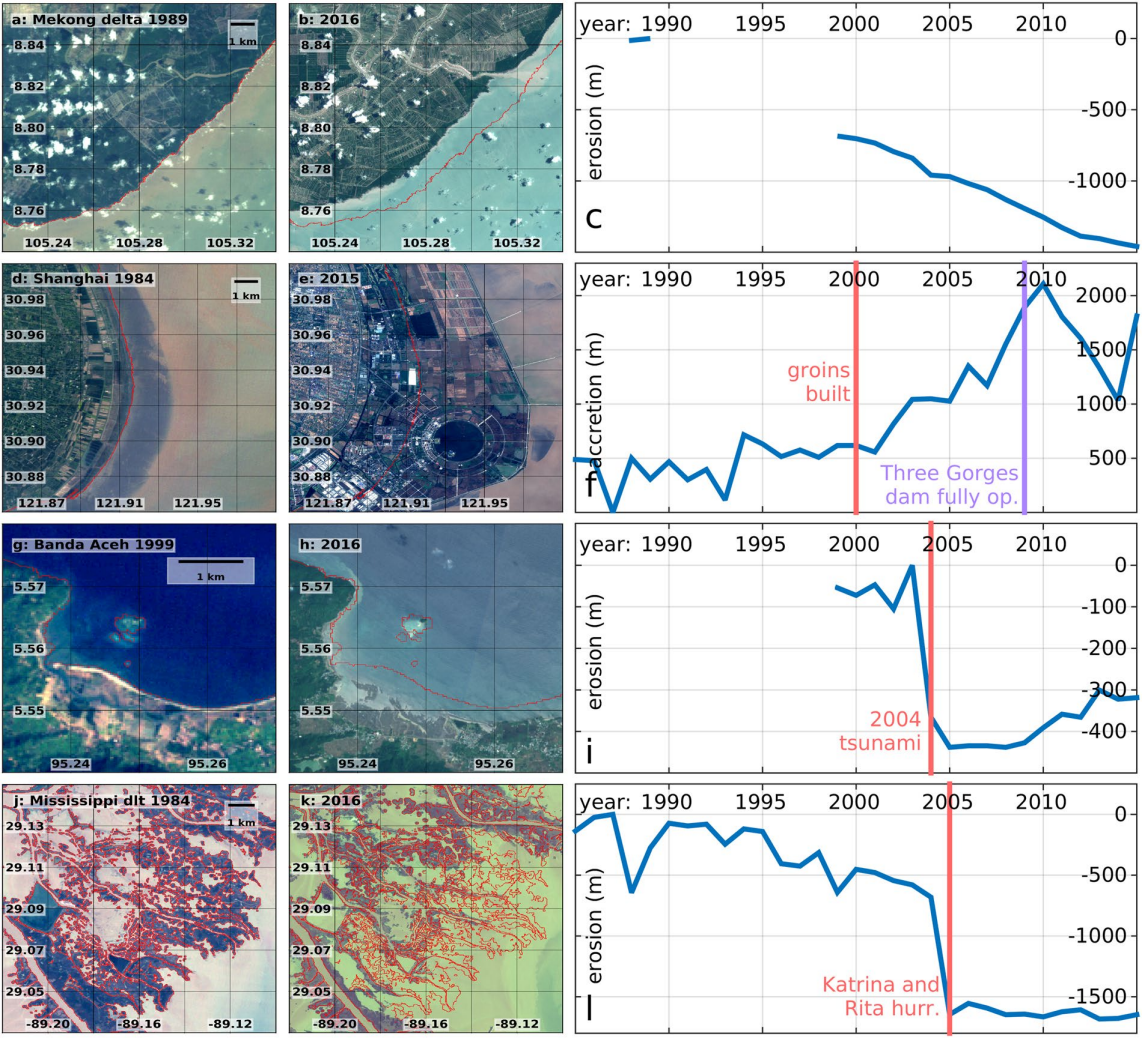
Satellite images of locations characterized by strong morphological change, at the beginning and at the end of the considered period, and time series of the cross-shore erosion/accretion. Mekong delta **(a–c)**, Shanghai **(d–f)**, the city of Banda Aceh (Indonesia, **g–i**), Mississippi delta **(j–l)**. The red lines in panels abdeghjk mark the coastline in the first year of observations. In panels cfil the time of relevant events is marked with a red line. This figure was generated using data from the USGS (http://earthexplorer.usgs.gov/), Copernicus Sentinel data 2016–2017, the Google-Earth-Engine^26^, and the programming languages python and MATLAB.

**Figure 5:**
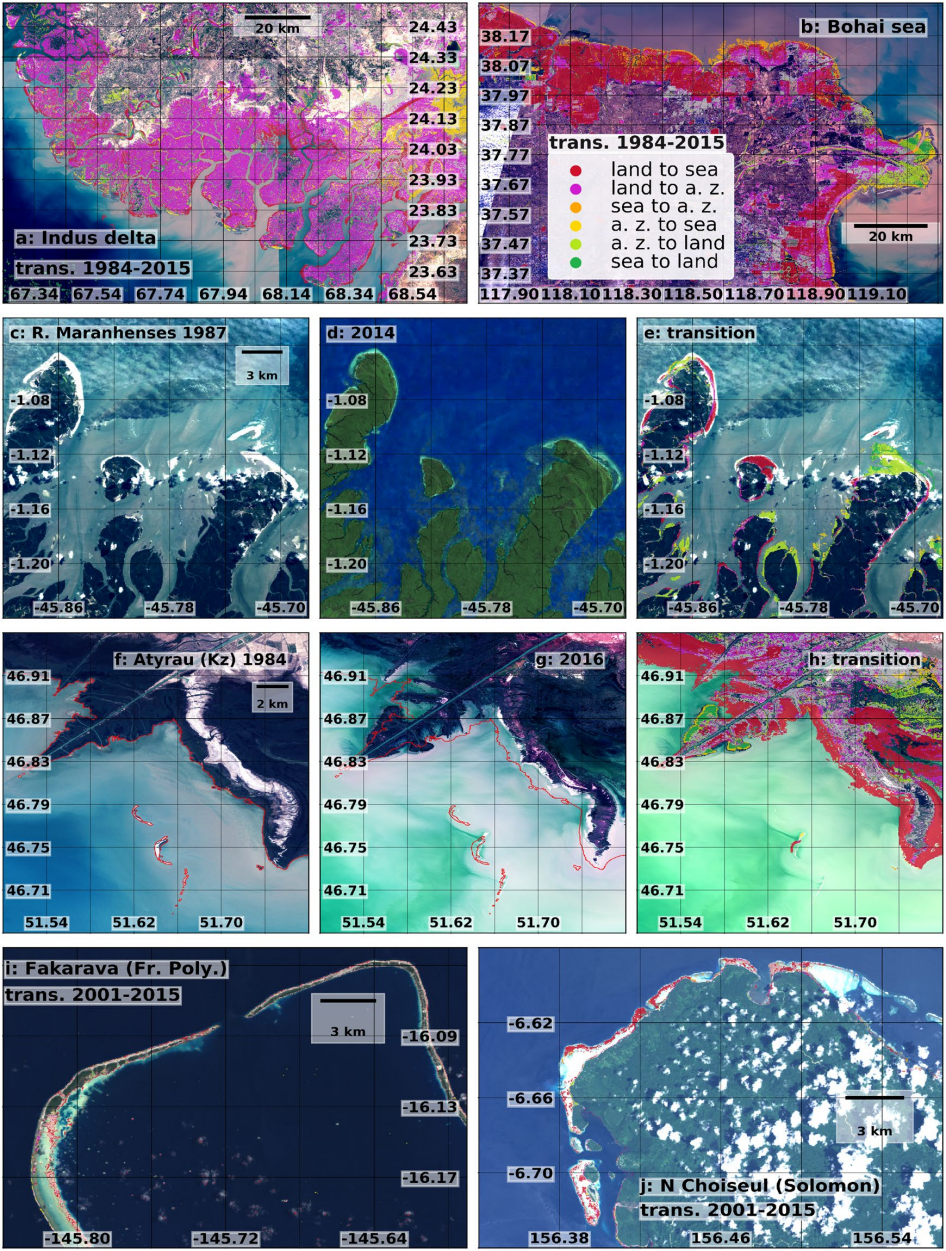
Satellite images of locations characterized by strong morphological change and maps of the observed transitions. Indus delta **(a)**, Southern Bohai sea **(b)**, the Reentrâncias Maranhenses (NE Brazil, **c–e**), the Fakarava island (French Polynesia, **f**) the Northern part of the Choiseul island (Solomon, **g**), Atyrau (Caspian sea, Kz, **h–j**). This figure was generated using data from the USGS (http://earthexplorer.usgs.gov/), Copernicus Sentinel data 2016–2017, the Google-Earth-Engine^26^, and the programming languages python and MATLAB.

Several major coastal transitions can be related with human activities that alter coastal systems directly or that can enforce or accelerate natural tendencies^31^. Dams are among the most prominent erosion factors, as they retain sediment that would normally feed the downdrift beaches^14,32^. Prominent examples are: (a) the delta of the Mekong river (Fig. 4a–c), along which several dams were installed in the last decades^23^; (b) the delta of the Indus river, at the border between India and Pakistan, associated with the exploitation of one of the world’s most extensive irrigation networks^33^, and where the largest erosion is revealed - exceeding 40 km of cross-shore retreat of the coastline (Figs 2c, 5a); (c) the estuary of the Sittaung river, in Myanmar, where hydropower plants have been installed^34^; and (d) the Brahmaputra delta^24^. For all the above cases, the effect of the upstream dams is combined with human degradation of the coastal mangrove forests, resulting in enhanced erosion due to the decreased capacity of the estuaries to retain sediments^12,15^. The decline of the mangrove forests is particularly strong at the Indus delta, where the extent of one of the world largest mangrove forests decreased by 72% between 1977 and 2006, increasing the vulnerability of this area to coastal erosion and hazards^33^. It should be mentioned that the degradation of mangrove forests is not always directly related with human activities, but can be also the result of intense natural disasters^35^.

The shores of the Bohai Sea in China constitute another prominent example of large-scale human intervention (Fig. 5b). The Yellow River is characterized by high sediment load^36,37^, induced since ancient times by human activities involving deforestation and cultivation on the Loess Plateau^38,39^. However in the last decades the sediment transport decreased, due to the installation of upstream dams and the increased water demand for human activities^32^. Moreover, the shifts in the course of the river at its estuary enhanced further the recent erosive trend in some areas^36^. This tendency was exploited in recent years through a large-scale conversion of previously dry areas into shrimp farming pods. As a result, the coasts south of Tientsin show the world’s second largest land loss, with a cross-shore land-sea transition exceeding 30 km (Fig. 2c), and the world’s largest transition from permanent land to permanent water (Fig. 1a of SI). The scale of human intervention in this area is also clear from the size of the Tientsin harbour. Another area of China where large-scale anthropogenic intervention enforced an existing trend is the coast south-east of Shanghai (Fig. 4d–f). This area, located in the Hangzhou Bay, between the deltas of the Yangtze and Qiantang Rivers, is characterized by a long term accretive trend that has been enforced since 2000, with the construction of “groins” trapping long-shore transported sediments. The time series of accretion in (Fig. 4c) shows a reverse trend after 2009, due to a partial re-inundation of some coastal areas. This may be associated with the activity of the Three Gorges Dam, the world largest dam, that started operating at full capacity on the Yangtze River from 2009^40^. It must be mentioned, however, that land reclamation continued at a sustained pace after 2009, in other parts of the Yangtze delta.

### Natural drivers

Natural processes can also induce strong morphological changes. For example, prominent erosion and accretion transitions are observed along the macro-tidal area of Reentrâncias Maranhenses, in north-east Brazil, close to the Amazon and Tocantins deltas. Here, the combination of oceanic waves and strong tidal currents created highly dynamic branched structures (Fig. 5c–e)^41^. The strong erosive effect of natural disasters such as tsunamis and storms is particularly visible in Banda Aceh in the northern coast of Sumatra, and at the mouth of the Mississippi river, in Louisiana. Banda Aceh is the major city that suffered the largest damages and fatalities from the 2004 tsunami^8^, which was the most intense ever registered. It resulted in about 400 m of erosion (Fig. 4g–i), with locally more than 1 km of permanent land loss close to the harbour (Figs 4, 5 of SI). The coast of Louisiana is one of the most exposed to tropical cyclones in the US^42^, and the rate of coastal erosion is strongly correlated with the intensity of the incoming extreme events. This is evident in the Pass-a-Loutre Wildlife Management Area, where beach erosion exceeded 1 km in 2005, the year of hurricane Katrina, the largest natural disaster in the history of the US^43^, and Rita (Fig. 4j–l).

**Figure 6:**
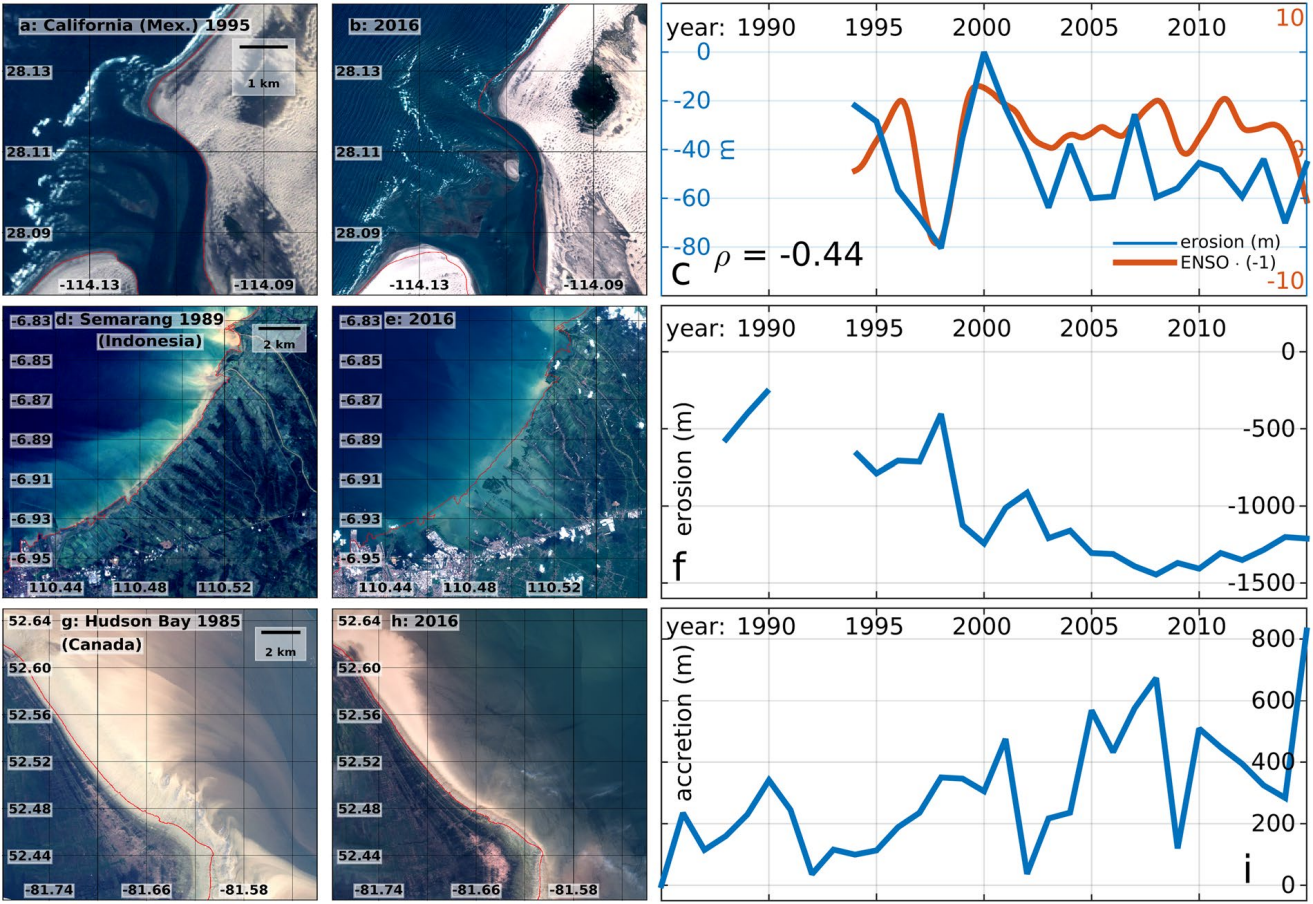
Satellite images of locations characterized by strong morphological change, at the beginning and at the end of the considered period, and time series of the cross-shore erosion/accretion. Mexican California (**a–c**), Semarang (Indonesia, **d–f**), Hudson Bay (Canada, **g–i**). The red lines in panels abdegh mark the coastline in the first year of observations. In panel c the ENSO index multiplied by (−1) is superimposed with the erosion series and their correlation coefficient *ρ* is indicated. This figure was generated using data from the USGS (http://earthexplorer.usgs.gov/), Copernicus Sentinel data 2016–2017, the Google-Earth-Engine^26^, and the programming languages python and MATLAB.

Storm frequency is in many areas correlated with the intensity of large-scale teleconnection patterns such as El Niño Southern Oscillation (ENSO). This explains the correlation between these patterns and the observed shoreline dynamics in some locations^7^. For example, in Guerrero Negro, in Mexican California, the evolution of the sandy shore after 1994 is significantly correlated with the ENSO index (*ρ = −0*.*44*, more than 95% significant, Fig. 6a–c), as erosion usually more intense under El Niño conditions, when storms are more frequent, and La Niña typically favors beach recovery^44^. Climate projections show that such teleconnection patterns will intensify^45–47^, generating more extreme waves and storm surges, and consequent coastal erosion.

### Relative Sea Level Rise

Intensified climate extremes combined with RSLR pose an increasing threat for future coastal security due to the complex and non-linear relationship between RSLR and erosion^48,49^. RSLR has been happening at an accelerated rate^50,51^, a trend projected to continue during the present century due to global warming^52–55^. The potential effects of RSLR can already be observed in areas undergoing strong land subsidence, such as in Semarang (Indonesia), where subsidence rates amount to 10 centimeters per year largely due to extensive groundwater extraction^56^. This has caused erosion in the order of 1.5 km close the city (Fig. 6d–f) in the last decade, thus also increasing its exposure to coastal hazards. The morphological activity along the Caspian coasts is related with changes of Caspian Sea Level (CSL), mainly due to the variability of precipitation over the Volga River basin. These changes consisted of a rapid rise until 1995 (in the order of 10 centimeters per year) and then a decrease^57,58^, resulting in intense morphological variations that are particularly strong along the Russian and Kazak coasts (Fig. 5f–h). RSLR further affects several low-lying “cays” (small, low-elevation, sandy islands), like the ones of Solomon, Kiribati, Tonga, French Polynesia, Vanuatu, Fiji Islands and other Small Island Developing States (SIDS), where the coastal coral reefs are being extensively eroded (Fig. 5I,j). Some of these SIDS are located in areas where RSLR is particularly strong, like Taro in the Solomon islands (RSLR ~1 centimeters per year, Fig. 10 of SI)^50^, where the capital of the Choiseul province will have to be relocated due to the increasing coastal erosion (Fig. 5j)^59^, or the Torres islands, in the Vanuatu archipelago^60^.

On the other hand, coasts subject to strong land uplift are generally accretive. This is partly the cause of the significant land gain in the Hudson Bay, in Canada, an area characterized by strong glacial isostatic rebound^61^, as well as by the confluence of several rivers (Fig. 6g–i).

### Implications

Our results reveal that anthropogenic factors are prominent drivers of global coastal morphological changes. This can be intentional by the expansion of water resources (e.g. for aquaculture), land claim (e.g. artificial islands), or the exploitation of structures (e.g. ports), sometimes taking advantage of existing tendencies. Many changes, however, are unforeseen or neglected side effects of human activities in coastal areas or in upstream major rivers catchments. The consequent degradation of coastal environments increases the vulnerability of coasts to wave activity and extreme events such as tropical cyclones. This, combined with an acceleration of RSLR and an intensification of extreme weather under global warming, could amplify the increasing erosion trend observed along the coasts of all oceans over the last three decades. Effective coastal planning and timely adaptation strategies are needed in order to halt this trend and to reduce coastal risks. Increasing coastal protection is likely to be cost-effective in well-populated high-income regions due to the potential large reduction in impacts, yet the required economic investments for artificial measures might not be available in lower income countries. Shoreline stabilization could be achieved at a lower cost through revegetation, and by safeguarding coastal ecosystems, such as marshes and reefs, that contribute to wave attenuation, sediment capture, and that self-adapt to water level^62–64^. Ultimately, in some places more drastic measures, such as the strategic relocation of structures and people or the abandonment of land may prove to be the only sustainable solution^65^. The presented dataset is freely available, in order to offer a range of opportunities to coastal researchers, managers and stakeholders, and includes globally consistent, high-resolution shoreline observations which can provide a deeper understanding of coastal dynamics, validation of models, and generation of robust risk profiles^66^. Moreover, the availability of accessible geospatial global data will assist data-driven and context-aware coastal planning, as well as adaptation measures.

## Methods

The global scale study of coastal evolution described in this manuscript is based on the analysis of the high-resolution Global Surface Water Explorer (GSWE) database^29^, that describes on a pixelwise basis the history of water presence during 32 years (1984–2015). In GSWE, each 30 m pixel is classified as: (a) “permanent water” if all the valid satellite observations on that pixel detect presence of water, which in the context of the present study corresponds to sea; (b) “land” if no observation is identified as water; (c) “seasonal water” if, within a year, some observations are identified as water and others as land, which in this study defines the active zone, and can be part of the intertidal area, but can also be areas that are occasionally (or quite often) inundated, for example as a result of the action of waves, or due to different river regimes in estuarine zones. Therefore, the active zone corresponds with the “seasonal water” bordering the sea, while the land pixels correspond to coastal areas. Each pixel can change state from year to year, passing, for example, from land to sea or from sea to land. Such transitions correspond, respectively, to a loss or a gain of land.

The analysis of transitions of permanent land and active zone represents an alternative approach to the explicit extraction of the intertidal area and coastal topography proposed in other studies^67^. The main advantages of the method applied here are: a) the explicit extraction of the intertidal area at specific times usually involves using models to estimate the water level, adding further sources of uncertainty apart from those related with satellite observations; and b) the active zone generally coincides with the intertidal area, but also includes areas frequently inundated by causes other than tides (e.g. waves or river floods in estuarine zones), that should be taken into account when considering coastal erosion and accretion in terms of land availability for human uses or terrestrial ecosystems. The main disadvantage of the approach described here is that it does not lead directly to a definition of coastline as a useful side-product. It only provides extents and shifts of land and active zone along given transects.

The relevant measures in this study are those of cross-shore erosion and accretion. Therefore, the first task of the analysis has been the development of a method to translate the pixelwise information of GSWE into these metrics. To this end, we defined a large set of virtual transects orthogonal to the coastline. Within each transect, we analyzed the space-time displacement of the border between land and water. The overall orientation of the coastline needed to define the location and orientation of the virtual transects was defined using the OpenStreetMap^68^ coastline data. The coarse spatial resolution of about 10 km of this dataset is sufficient for that specific purpose. The produced transects were used in combination with the GSWE dataset at 30 m resolution to determine the location and extent of accretion and erosion areas around the globe. It should be mentioned, that while such a resolution is dictated by the underlying imagery, often meaningful estimations can be found on lower scales in cross-shore direction (e.g. 10 m) thanks to long-shore averaging between neighboring pixels (e.g. Fig. 10 of SI).

Transects were generated at a spacing of 250 m, resulting in a total of *N*_*t*_ = 2,142,679 virtual transects. The land-water transitions were analyzed on 200 m wide stripes surrounding each transect in order to guarantee a good coverage of the coastal surface. In order to account for the complex morphology of the coastline, each transect has a variable length and was generated in such a way that one of its extremities lies on “permanent” sea and the other on “permanent” land. Residual erroneous transects (about 2.4% of the total number of transects, Fig. 12 of SI) are mainly located in sheltered areas, like the internal parts of fjords or river deltas, or areas sheltered by the presence of many islands, where coastal erosion is not expected to play a major role, or in locations where water transition data from GSWE are unavailable, such as for a few small islands.

The analysis along the transects of the transition surfaces obtained from GSWE, i.e. the pixelwise information about the transitions dry-wet and wet-dry, enabled determination of the location of the land-water transitions observed between 1984 and 2015. Six different categories of transitions have been characterized. Three relate to coastal erosion: permanent land to permanent sea (indicated with the symbol ${Y}_{ls}$), permanent land to active zone (indicated with the symbol ${Y}_{la}$), and active zone to sea (${Y}_{as}$). The three other transitions relate to accretion: permanent sea to permanent land (${Y}_{sl}$), permanent sea to active zone (${Y}_{sa}$), and active zone to land (${Y}_{al}$). The transitions were subsequently aggregated as in (1–3) in order to provide a balance of lost and gained land (*L*_*lost*_ and *L*_*gain*_) and a balance of lost and gained active zone (*A*_*lost*_ and *A*_*gain*_).

1 $${L}_{lost}={Y}_{ls}+{Y}_{la},$$

2 $${L}_{gain}={Y}_{sl}+{Y}_{al},$$

3 $${A}_{lost}={Y}_{as}+{Y}_{al}\,,$$

4 $${A}_{gain}={Y}_{sa}+{Y}_{la}\,.$$

The algorithm (implemented as a python application in Google Earth Engine^26^) consists of lengthwise slicing the surface around each transect ${\theta }_{i}$ into stripes with length *l* = 60 m (Fig. 13 of SI) along the transect. Then, starting from the seaward side of the transect, it computes the intersection ${s}_{ij}^{k}$ between each slice ${S}_{i}^{k}$ and each transition surface ${Y}_{j}$. The average length associated to the transition inside the slice is given by

5 $${l}_{ij}^{k}=l\cdot {s}_{ij}^{k}/{S}_{l}^{k}\,.$$

The iteration stops when a slice with more than 50% on permanent land is encountered. The total length of the transition ${Y}_{j}$ for the transect ${\theta }_{i}$ is then given by

6 $${Y}_{ij}=\sum _{k}{l}_{ij}^{k}\,.$$

For transects displaying a transition above 30 m we estimated a time series of yearly lengths of permanent land and active zone, which provides an indication on the yearly state of the coastline. For this purpose we studied the yearly surfaces of water occurrence from GSWE along each transect. Given transect ${\theta }_{i}$, its portion (with surface *T*_*i*_) spanning from its seaside extremity to the first point of all-time permanent land was considered. For each year *y* the portion *G*_*iy*_ of *T*_*i*_ covered by permanent land was estimated. The position of the coastline in the year *y* with respect to the all-time coastline is then given by

7 $${C}_{iy}={G}_{yi}/W\,,$$

where *W* is the average width of the transect (~200 m).

### Known issues and limitations

The availability of satellite data is not uniform for all the locations, and so the time horizon of the analysis is not everywhere 32 years (Fig. 14 of SI). Moreover, in some locations the scarce availability of satellite observations combined with poor observation conditions (e.g. frequent cloud or snow occurrence, or long polar night) do not allow a satisfactory determination of the land-water transitions. This occurs, for example, close to the poles and in some islands and cays for which fewer valid observations are available. Therefore, a set of criteria based on the number of valid observation available, has been set up to identify and filter out these locations (excluding, for example, areas with an insufficient number of valid observations). As a consequence, the coverage of the global coast is incomplete: only about 86% of the coastline at latitudes below 63 degrees (Fig. 14 of SI).

Another limitation of the study relates to the spatial (30 m) and temporal (8-day cycle or 16 days when two satellites operate concurrently) resolutions of the satellite imagery, which prevent capturing small-scale or short terms changes.

A limitation associated with the definition of virtual cross-shore transects close to the angles of coastline is that at the convex side of the angles transects are superimposed, leading to an overestimation of the transition surface when transects are summed. At the concave side, on the other hand, transects do not cover entirely the land or sea surface, leading to underestimation of the transition surface. The statistical error associated with this geometrical problem can be evaluated for each coastal segment as the quadratic sum of the approximate sizes of the superimposing or missing transition surfaces (see Section 2 of SI for details). While this limitation can lead to relevant uncertainty of local estimates of the transition surface, the error drops below 1% when the transitions are averaged on continental scales, because the statistical error of the mean on N coastal segments scales approximately as $1/\sqrt{N}$.

### Validation

The accuracy of this study depends strongly on the accuracy of the data provided by GSWE, which has been extensively validated^29^. A large-scale validation of the present dataset on shoreline dynamics is hampered by the scarcity and heterogeneity of field measurements. We compared our results versus 8 different independent studies that cover 12 areas at different scales, showing good agreement (Section 1 and Table 1 of SI).

## Electronic supplementary material

Supplementary Information

## Data Availability

The data produced in this study are freely available at http://data.jrc.ec.europa.eu/collection/LISCOAST.
