# Supplementary material for: Global long-term observations of coastal erosion and accretion
Source: Sci Rep. 2018 Aug 27;8:12876. doi: 10.1038/s41598-018-30904-w (PMC6110794; doi:10.1038/s41598-018-30904-w)
Supplement: Supplementary file 1 — Supplementary Information [file 41598_2018_30904_MOESM1_ESM.docx]

Supplementary Information

**Global long-term observations of coastal erosion and accretion**

Lorenzo Mentaschi^1*^, Michalis Vousdoukas^12^, Jean-Francois Pekel^1^, Evangelos Voukouvalas^1^, Luc Feyen^1^

^1^European Commission, Joint Research Centre (JRC), Via Enrico Fermi 2749, 21027, Ispra, Italy

***^2^Department of Marine Sciences, University of the Aegean, Mitilene, Greece***

***^*^Corresponding author: lorenzo.mentaschi@ec.europa.eu***


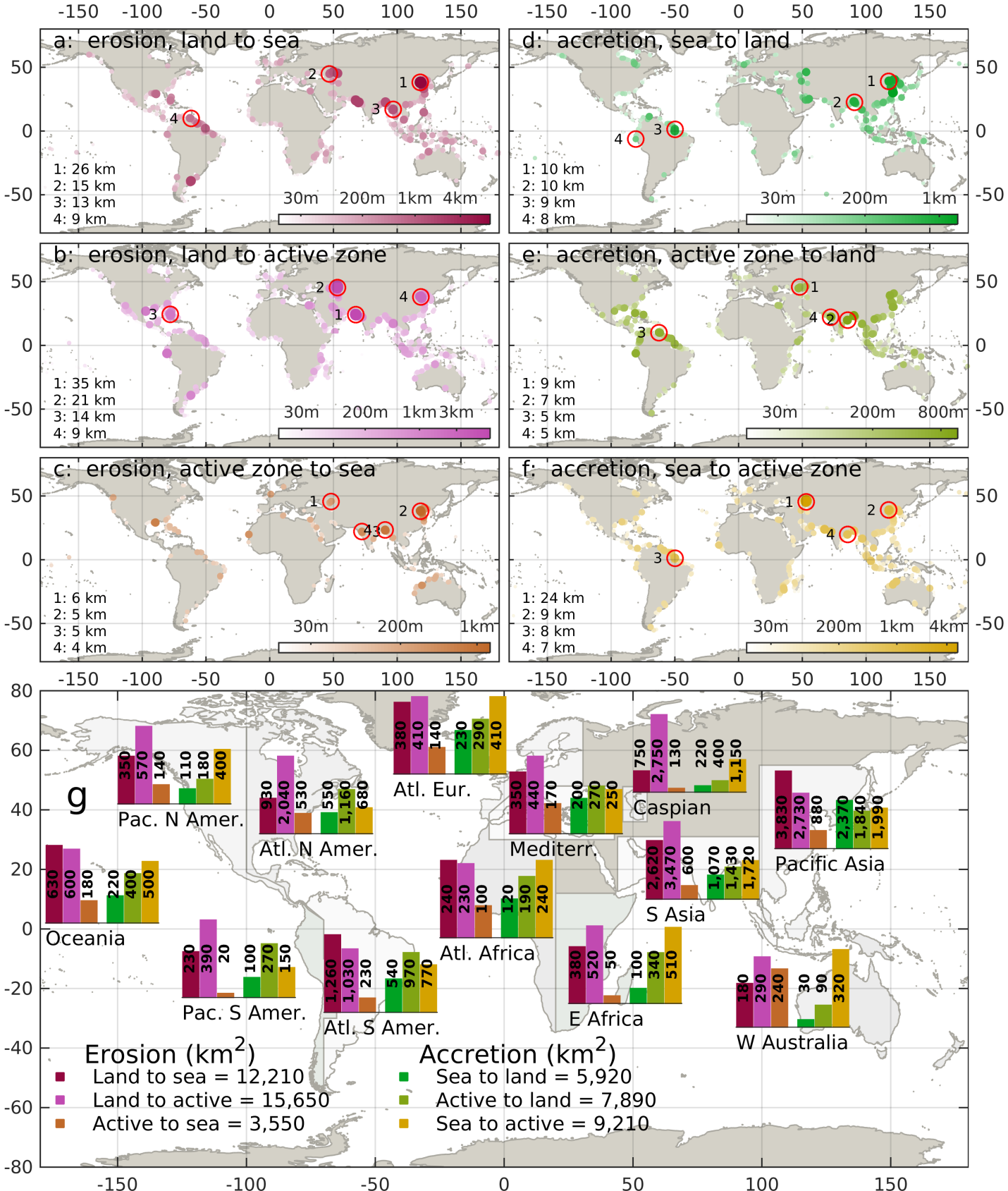


Figure : Transitions: land to sea (panel a), land to active zone (b), active zone to sea (c), sea to land (d), active zone to land (e), sea to active zone (f) expressed in coast-normal meters of erosion and accretion aggregated on coastal segments of 100km. In panels a, b, c, d, e, f the 4 spots with the highest local transition are also evidenced. Overall transitions (panel g), aggregated per continent/ocean and globally. This figure was generated with the MATLAB programming language

## Comparison with local studies

A large-scale validation of our measurements is hampered by the scarce availability of field measurements, and by their heterogeneity in terms of time horizon, space extension (single numbers are generally provided for extended areas), and types of measure (cross-shore length, erosion/accretion surface, surface per year, approximate diameter of lost/gained islands, the difference between permanent land and active zone is generally not considered …). This underlines the importance and usefulness of a global dataset of space-time consistent and homogeneous measurements of coastal morphodynamics. On the other hand, local studies are usually developed paying attention to local conditions (such as local dynamics, as well as small local features) and are therefore characterized by a higher accuracy than achievable in a global study. It is therefore important to provide a comparison of our results with the estimations of local studies at specific locations. This comparison is presented in Table 1. The agreement is good for all examined studies and this shows that our results are locally relevant. Each of the comparisons have particular specificities that are listed below.

- Though the time horizon of the measurements of ^1^ for the Bohai sea is significantly different from that of this study (1976-2000 vs 1984-2015), their observations of cross-shore erosion for Daikou and Shenxiangou promontories are fairly consistent with ours. At the Quingshuigou promontory our measurements underestimate the cross-shore accretion and are affected by relevant geometrical error. This is mainly due to the direction of the accretion that is not normal to the coastline used in this study (Figure 2).
- There is a good agreement between our estimation of permanent land loss in Sandwip Island (Bangladesh, Figure 3) and the one of eroded surface provided by ^2^. The discrepancy in the measurements of gained permanent land can be partly explained by the different time horizon (that can involve big changes in this highly active area). On the other hand we distinguish between permanent and active zone, that ^2^ does not. If we sum the observed accretion of permanent land with the accretion of active zone, we obtain figures very close to ^2^ (55 vs 54 km^2^).
- In Banda Aceh in a transect close to the harbor (Figure 4), we find between 2002 and 2005 a cross-shore loss of permanent land of about 1.1 km (Figure 5), which is in the same order of the observations by ^3^ (1.5 km). The distribution of the transition in 2 years instead of 1 is due to the low number of valid satellite observations available for 2002, 2003 and 2004.
- Our estimations of erosion/accretion in km^2^/year for the three subareas of the Mekong delta are within the ranges measured by ^4^ between 2003 and 2012.
- At Atchafalaya mouth (Mississippi, USA) our measurements of the surface of accretion are in good agreement with the ones provided by ^5^ (55 km^2^ when summing new land and new active zone, versus 59 km^2^ measured by 5), though our estimate is affected by relevant geometrical error, especially at the eastern mouth (Figure 6).
- Our estimate of cross-shore accretion at Palm Jumeirah (Dubai, Figure 7) is in full agreement with the diameter of the artificial structure indicated by ^6^. The difference between the two lengths is due to the presence of water within the structure that is not accounted for as accretion in our study.
- At the mouth of Chann creek in the Indus delta, our estimate of cross-shore land loss is close to the one provided by ^7^, though the time horizons of the two studies are consistently different, and even if our cross-shore direction is relative to a low resolution coastline, while the distance used in ^7^ is the diameter of an island at the mouth that was completely eroded.
- At the Narrabeen beach (Sydney, Australia), our estimates of yearly cross-shore beach length were compared with the time series provided by ^8^ at transect PF1, showing good agreement (Figure 9).

| Location | | Time horz. | | Land balance | Active zone balance | Ref. time horizon | Ref. measure | Ref. |
| --- | --- | --- | --- | --- | --- | --- | --- | --- |
| Daikou (Bohai sea) | 1984-2015 | | 6.6±0.1 km eros. | | 1.7±0.05 km acc. | 1976-2000 | 7 km erosion | 1 |
| Shenxiangou  (Bohai sea) | 1984-2015 | | 4.1±0.4 km eros. | | 1.5±0.1 km acc. | 1976-2000 | 4.5 km erosion | 1 |
| Qingshuigou  (Bohai sea) | 1984-2015 | | 0.8±1.5 km eros. | | 3.5±1.6 km acc. | 1976-2000 | 7 km accretion | 1 |
| Sandwip (Bangladesh) | 1988-2015 | | 59±8 km^2^ eros.  41±11 km^2^ accr. | | 37±7 km^2^ eros.  14±7 km^2^ accr. | 1989-2010 | 57 km^2^ eros.  54 km^2^ accr. | 2 |
| Banda Aceh (Indonesia) | 2002-2005 | | 1.1 km max eros. | | - | 2004 | 1.5 km max eros. | 3 |
| Mekong  (Delta Ditrib. Mouths) | 1988-2015 | | 0.5±0.04 km^2^/yr accr. | | 1.3±0.04 km^2^/yr acc. | 2003-2012 | 0.26 – 0.78 km^2^/yr accr. | 4 |
| Mekong  (Southern Chinese Sea) | 1988-2015 | | 2.1±0.02 km^2^/yr eros. | | 0 km^2^/yr | 2003-2012 | 2.0 – 2.7  km^2^/yr eros. | 4 |
| Mekong  (Gulf of Thailand) | 1988-2015 | | 0.6±0.06 km^2^/yr eros. | | 0.1±0.06 km^2^/yr eros. | 2003-2012 | 0.57 - 0.87  km^2^/yr eros. | 4 |
| Mississippi  (Atchafalaya mouth) | 1984-2015 | | 21±3.3 km^2^ accr. | | 34±6.4 km^2^ accr. | 1989-2010 | 59 km^2^ accr. | 5 |
| Palm Jumeirah (Dubai) | - | | 4.5 km max accr. | | - | - | ~5 km  island diameter | 6 |
| Chann Creek  (Indus delta) | 1987-2015 | | 1±0.01 km eros.  land to sea | | 1.1±0.01 km  land to a.z. | 1976-2007 | 1 km erosion | 7 |
| Narrabeen beach, transect PF1  (Sydney, Australia) | 1987-2015 | | ~20 m accr.  (see Figure 9) | |  | 1987-2015 | ~20 m accretion  (see Figure 9) | 8 |

Table : Erosion and accretion estimated for land and active zone, compared with the measurements provided by existing studies. The geometrical error related with our estimates is also indicated.


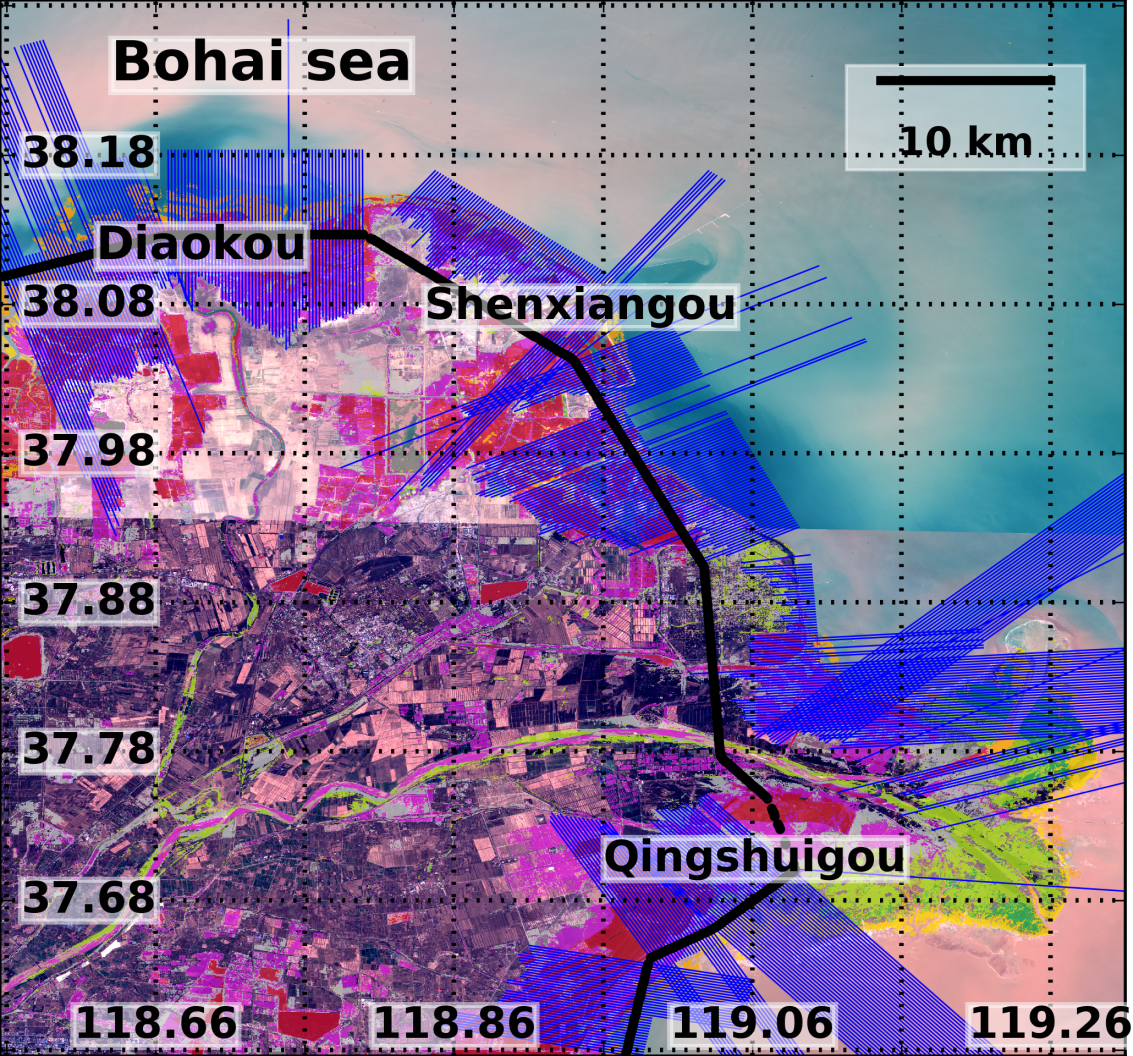


Figure : Transects and transitions in Southern Bohai Sea. The colors of the transitions are like in Figure 3 of the manuscript. The black broken line is the low-resolution coastline. This map was produced using data from the USGS (http://earthexplorer.usgs.gov/), Copernicus Sentinel data 2016-2017, the Google-Earth-Engine^9^, and the python programming language.


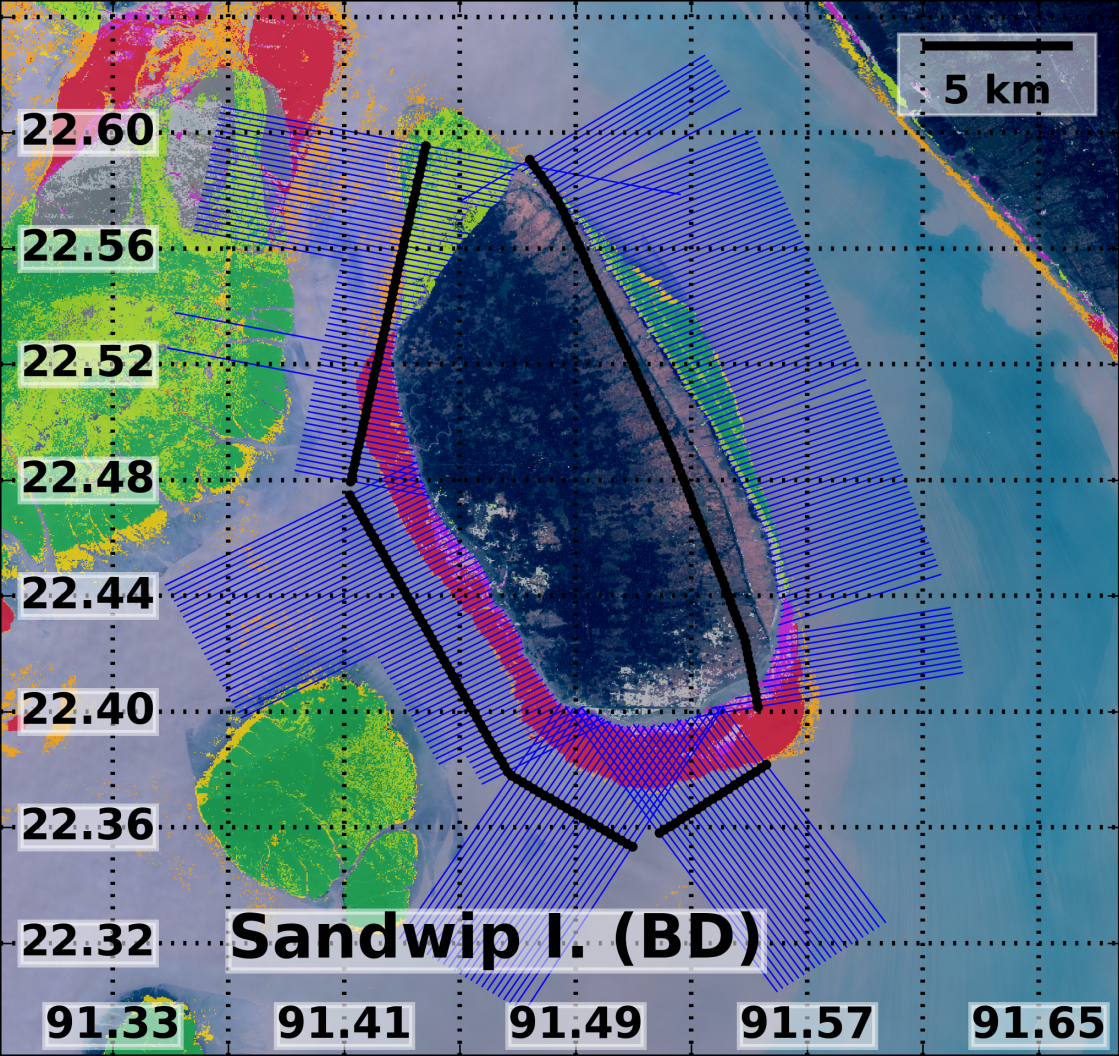


Figure : Transects and transitions at Sandwip Island (Bangladesh). This map was produced using data from the USGS (http://earthexplorer.usgs.gov/), Copernicus Sentinel data 2016-2017, the Google-Earth-Engine^9^, and the python programming language.


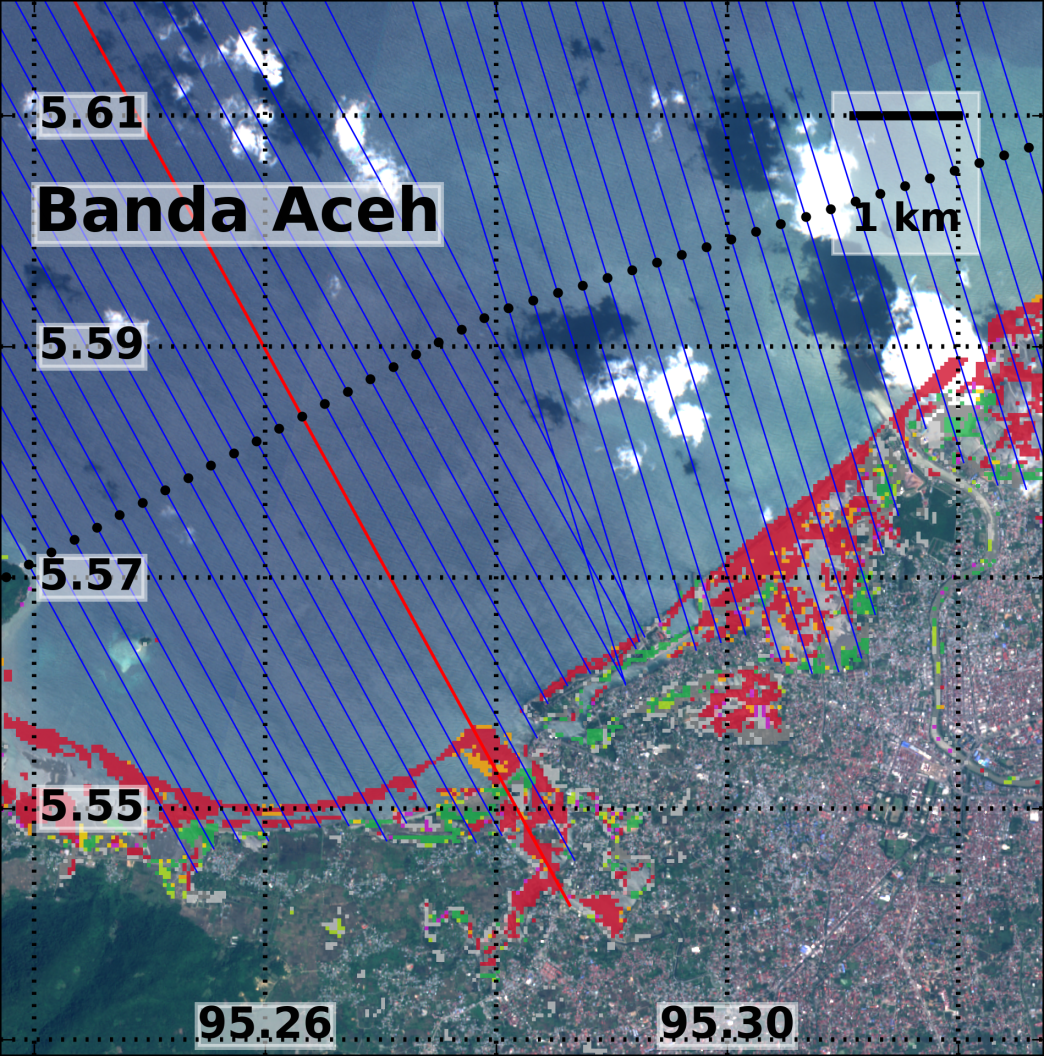


Figure : Transects and transitions in Banda Aceh, Indonesia. The transect with the longest observed transition is marked in red. This map was produced using data from the USGS (http://earthexplorer.usgs.gov/), Copernicus Sentinel data 2016-2017, the Google-Earth-Engine^9^, and the python programming language.


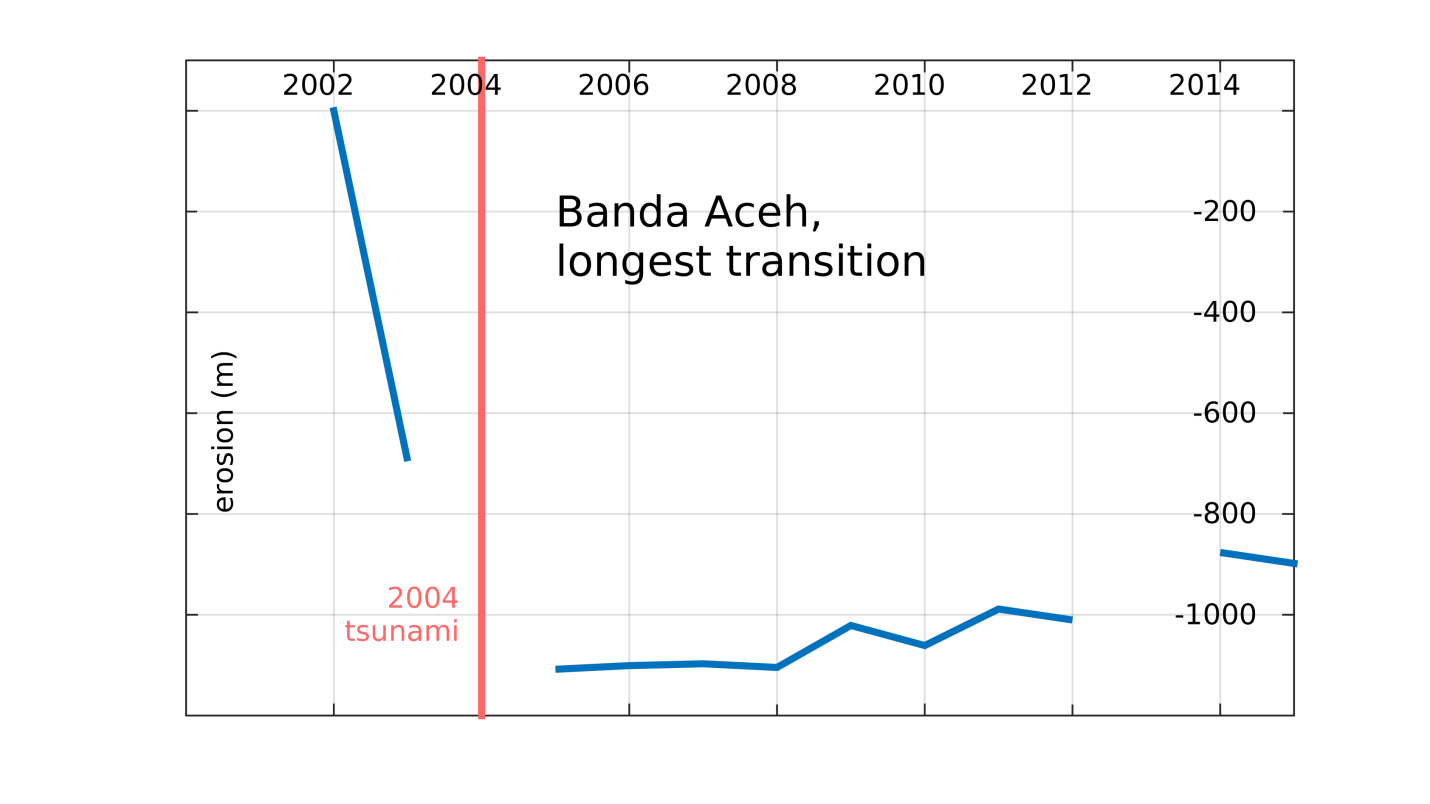


Figure : Time series of the longest transition observed in Banda Aceh. This figure was generated using the python programming language.


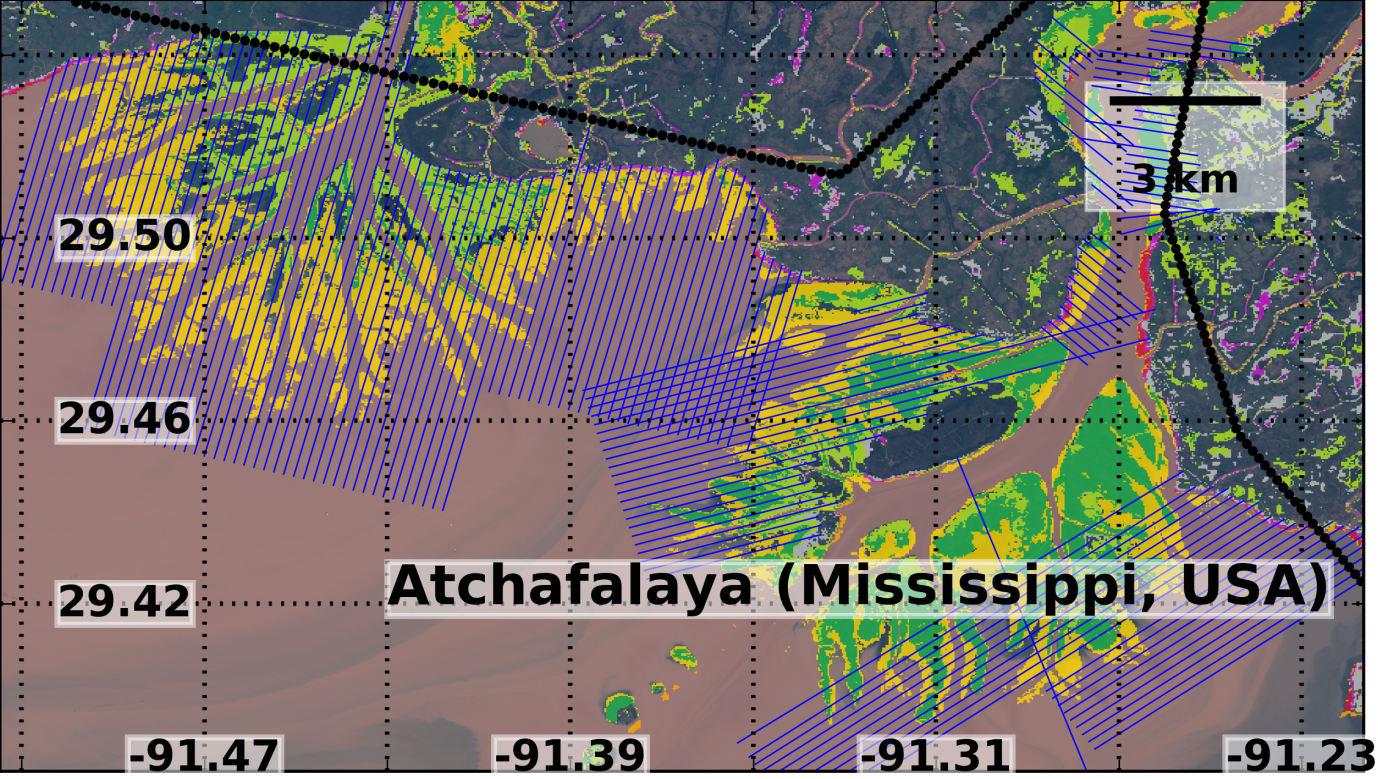


Figure : Transects and transitions at Atchafalaya mouth (Mississippi delta). This map was produced using data from the USGS (http://earthexplorer.usgs.gov/), Copernicus Sentinel data 2016-2017, the Google-Earth-Engine^9^, and the python programming language.


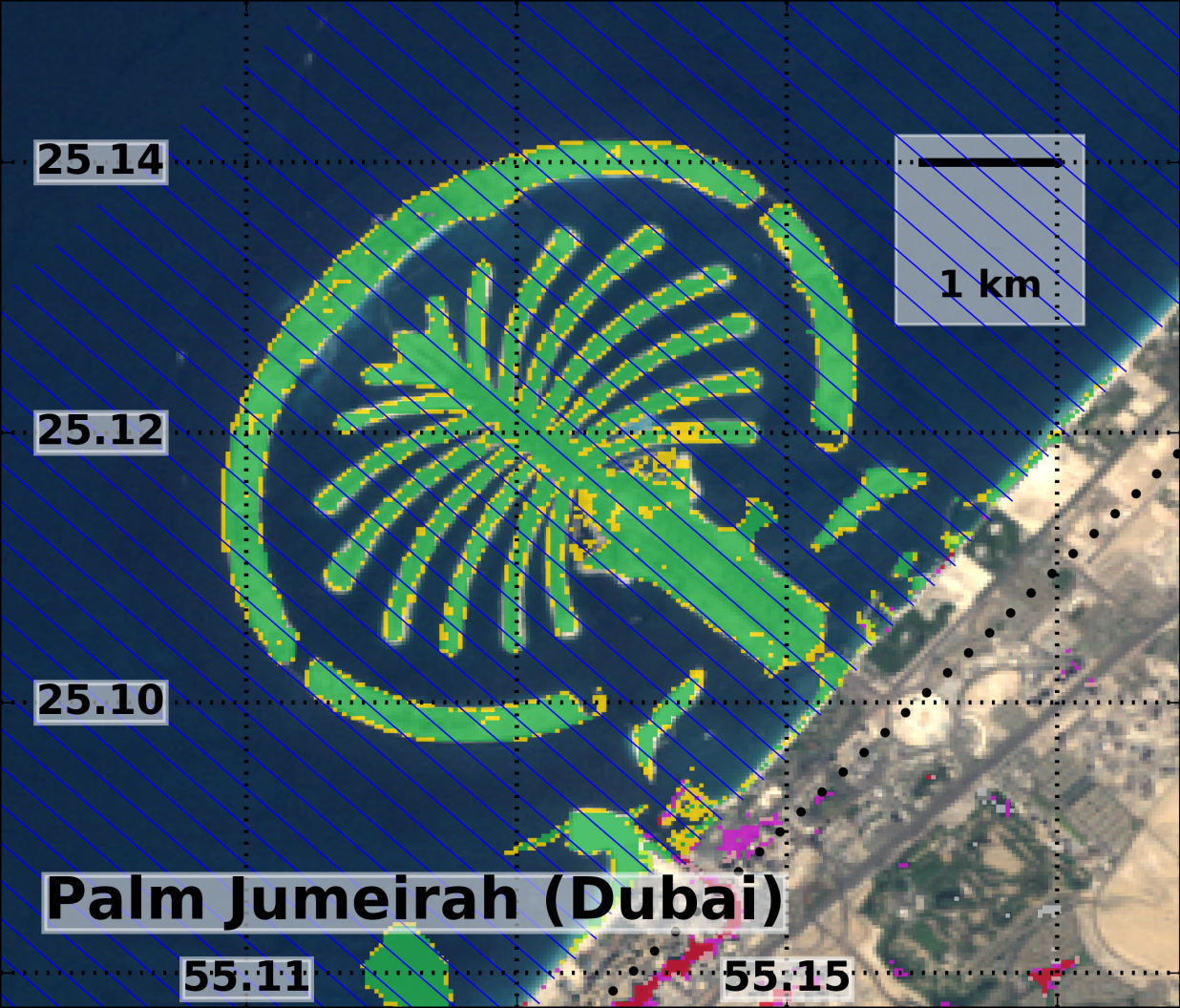


Figure : Transects and transitions at Palm Jumeirah, Dubai. This map was produced using data from the USGS (http://earthexplorer.usgs.gov/), Copernicus Sentinel data 2016-2017, the Google-Earth-Engine^9^, and the python programming language.


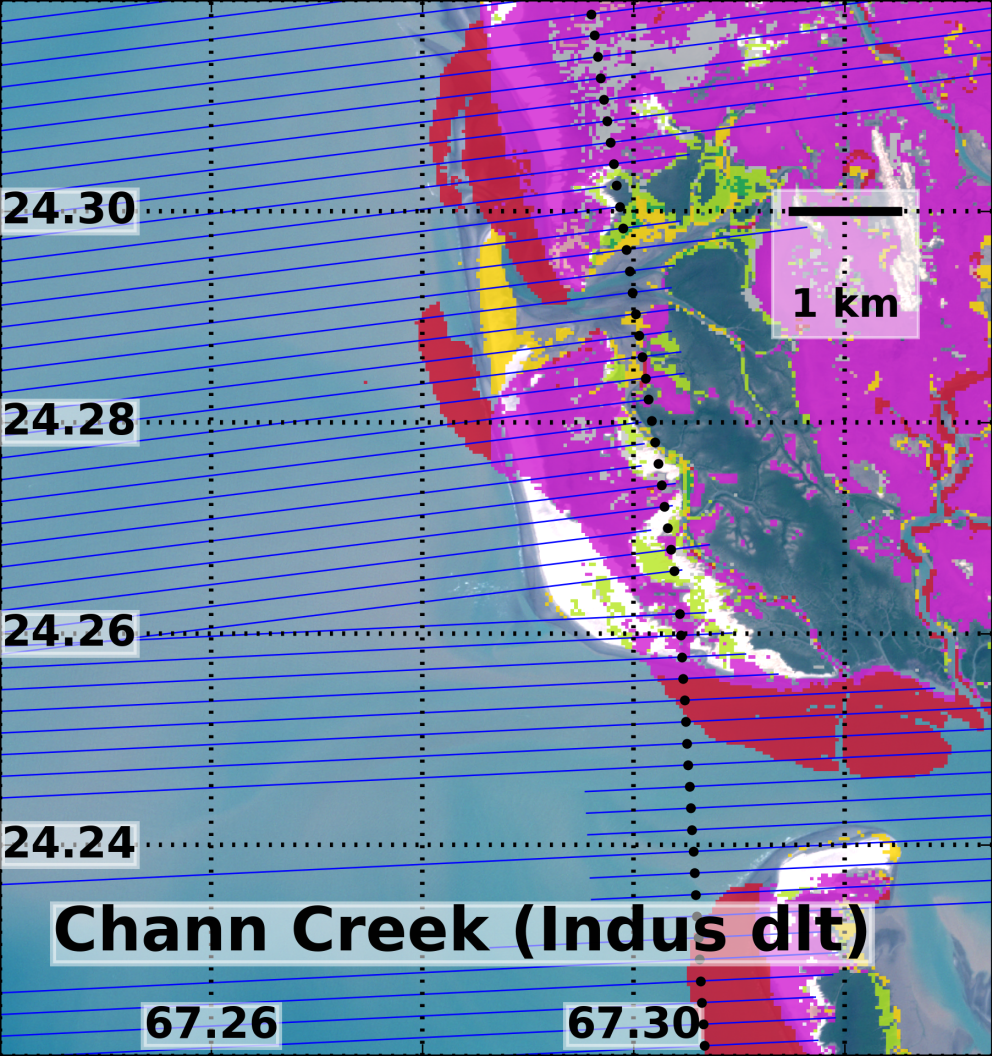


Figure : Transects and transitions at Chann Creek, Indus Delta. This map was produced using data from the USGS (http://earthexplorer.usgs.gov/), Copernicus Sentinel data 2016-2017, the Google-Earth-Engine^9^, and the python programming language.


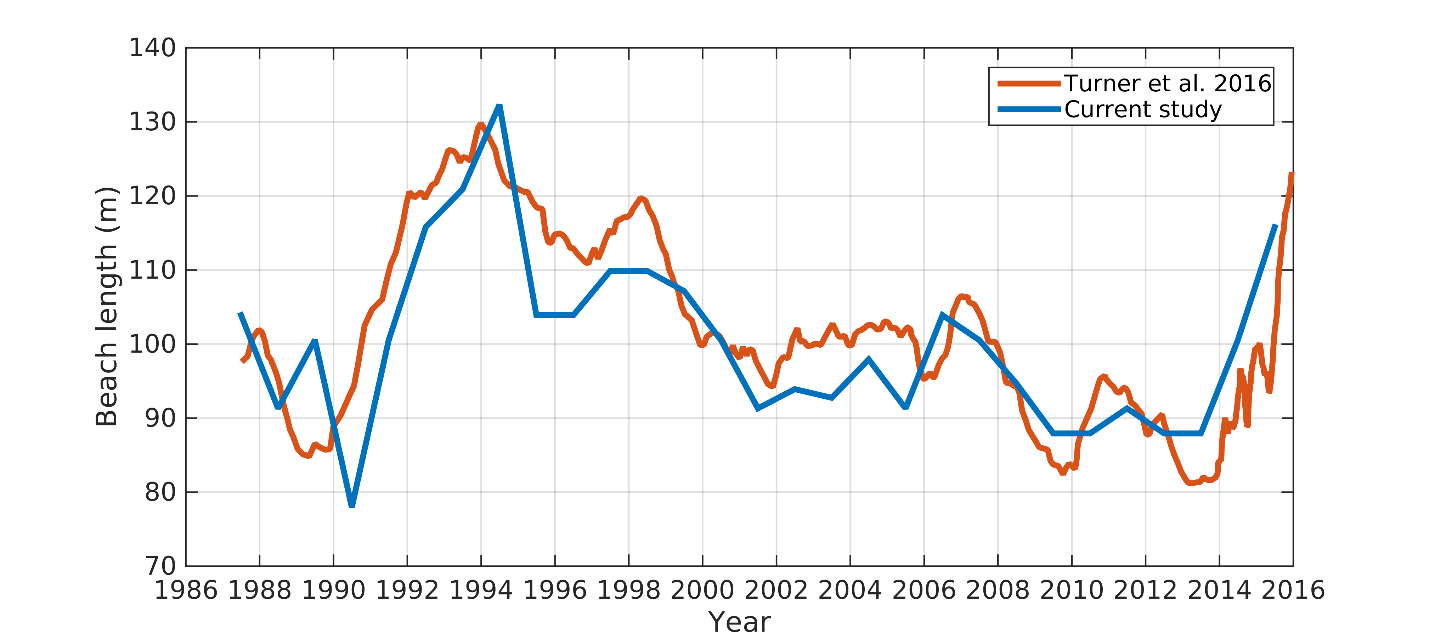


Figure : Narrabeen beach, Australia: comparison between the yearly beach length estimated in this study versus the one measured at transect PF1 by ^8^, from 1987 to 2015. This graph was created using the data provided by ^8^ and the MATLAB programming language.


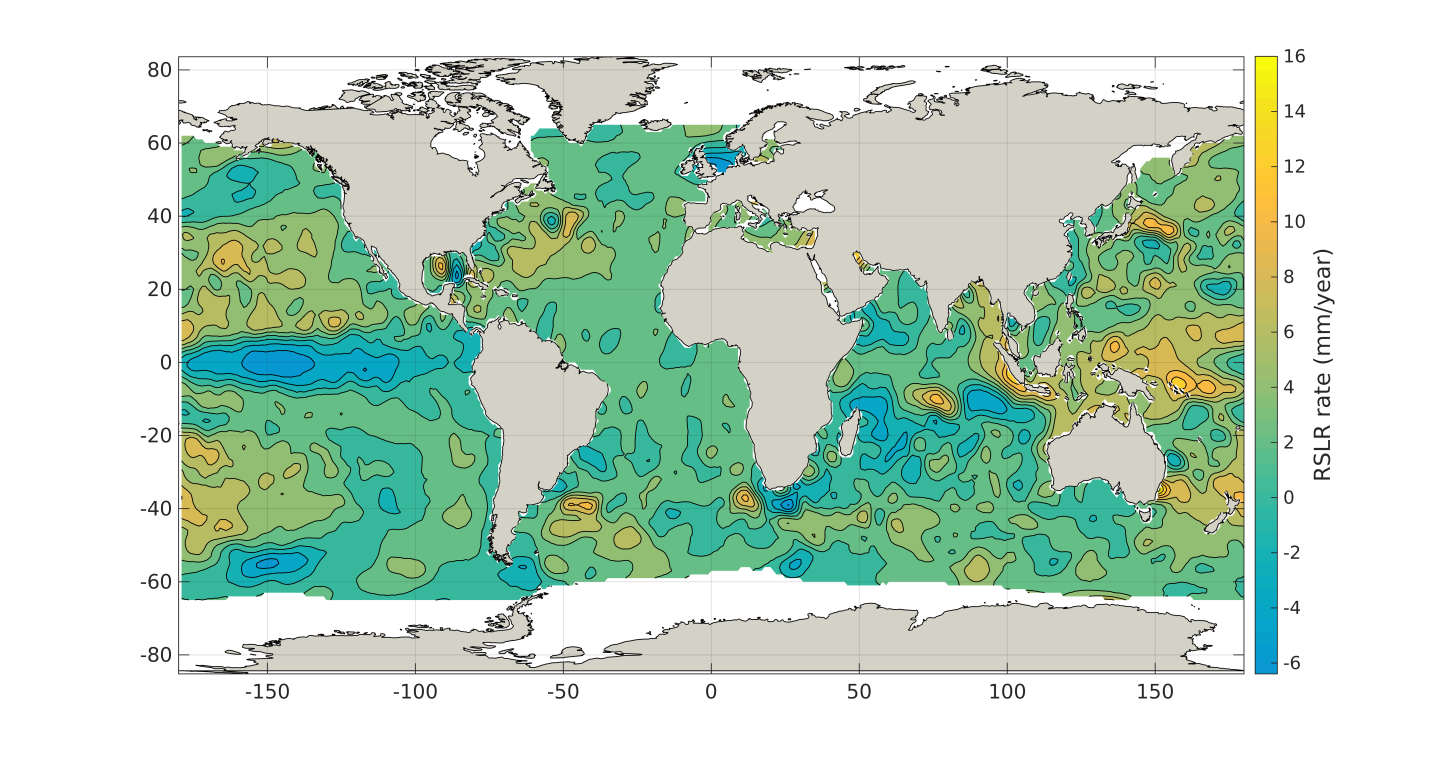


Figure : Relative Sea Level Rise (RSLR) rate in mm/year as estimated by^10^. This figure was generated with the MATLAB programming language.

## Geometrical error

Defining cross-shore virtual transects and summing/averaging the transitions along them may introduce a certain degree of uncertainty of geometrical nature in some locations. The main source of error are along transects close to the angles between coastline segments that intersect each other. At the convex side of the angle the transects are superimposed, leading to an overestimation of the transition surface when the transects are summed. At the concave side the transects do not cover entirely the land/sea surface, leading to an underestimation of the transition surface (Figure 10).

A measure of the error associated with local sums/average of coastal transitions can be obtained by the approximate surface of superimposed/missing areas. Let us consider a set of transects $\theta_{j}$ covering two neighboring segments of the coastline. The total surface associated with a transition *Y* on the two coastal segments is given by

|  | $S_{Y}=\sum_{j} D\cdot Y_{j} ,$ | 1 |
| --- | --- | --- |

where *D*=250m is the constant distance between two transects. We define the approximate superimposition/missing surface *S_e_* as (Figure 10)

|  | $S_{e}=\left\{\begin{matrix} Y_{ang}^{2}\cdot\tan\left(\frac{\pi-\vartheta}{2}\right) , \vartheta>\frac{\pi}{2} \\ Y_{ang}^{2} , \vartheta\leq\frac{\pi}{2} \end{matrix} ,\right.$ | 2 |
| --- | --- | --- |

where *Y_ang_* is the transition length of the transects close to the angle.

Let us consider a broken line of *N+1* coastline segments with *N* angles ϑ*_i_* between them. Then we can roughly consider the superimposition/missing surfaces $S_{e}^{i}$ as normally distributed deviations. Therefore, the quadratic error $\sigma_{surf Y}^{2}$ of the transition sum is given by the quadratic sum of the $S_{e}^{i}$:

|  | $\sigma_{surf Y}^{2}=\sum\left(S_{e}^{i}\right)^{2}=N\cdot\overline{S_{e}^{2}} .$ | 3 |
| --- | --- | --- |
|  |  |  |

The relative error on the surface sum is then given by

|  | $\sigma_{rel}^{Y}=\frac{\sigma_{surf Y}}{\sum S_{Y}}=\frac{\sqrt{N\cdot\overline{S_{e}^{2}}}}{N\cdot\overline{S_{Y}}}=\frac{1}{\sqrt{N}}\frac{\sqrt{\overline{S_{e}^{2}}}}{\overline{S_{Y}}} .$ | 4 |
| --- | --- | --- |

It is easy to show that the relative error on the average cross-shore transition is equal to the relative error of the transition surface. It is worth noting that while the error can be locally significant, when a coastal transition is summed over a large number of coastal segments it rapidly decreases like $1/\sqrt{N}$.


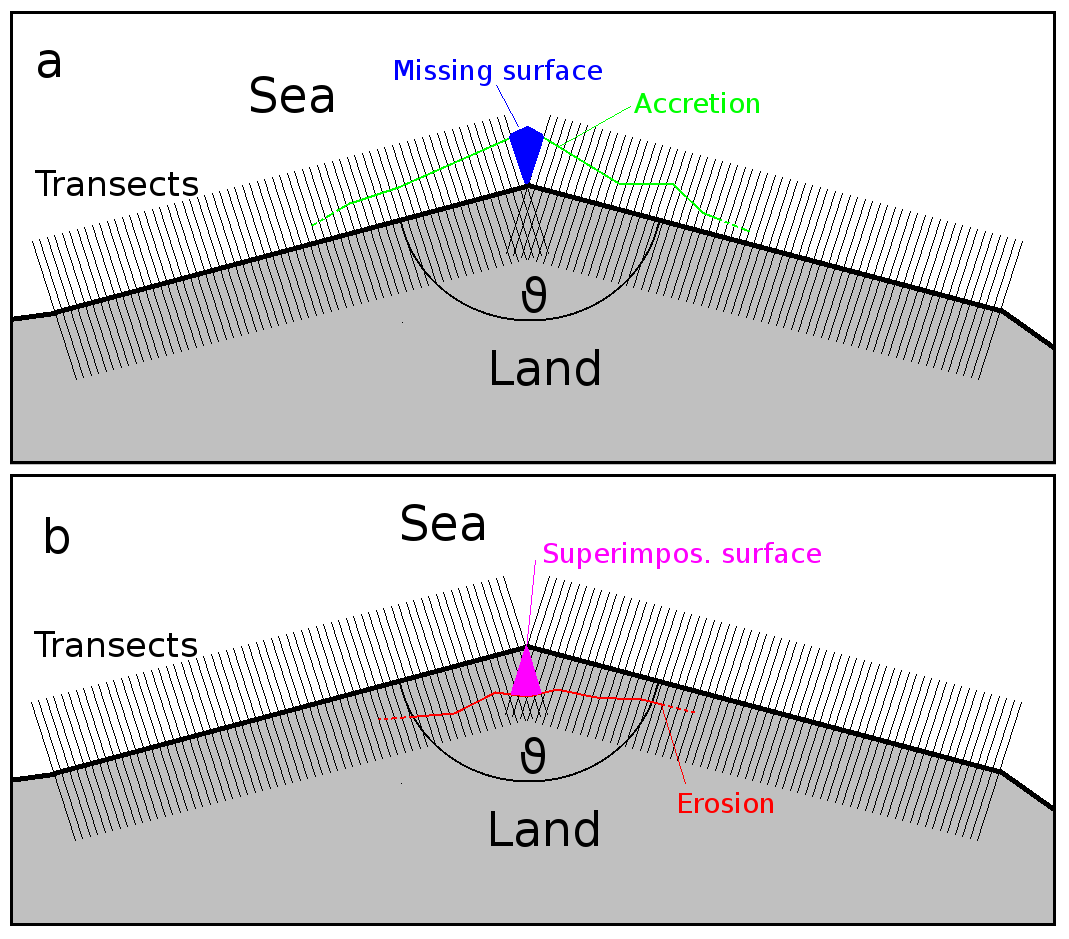


Figure 11: Erroneous superimposition/missing areas at the angle between two coastal segments. This figure was generated using the kolourpaint application.

**
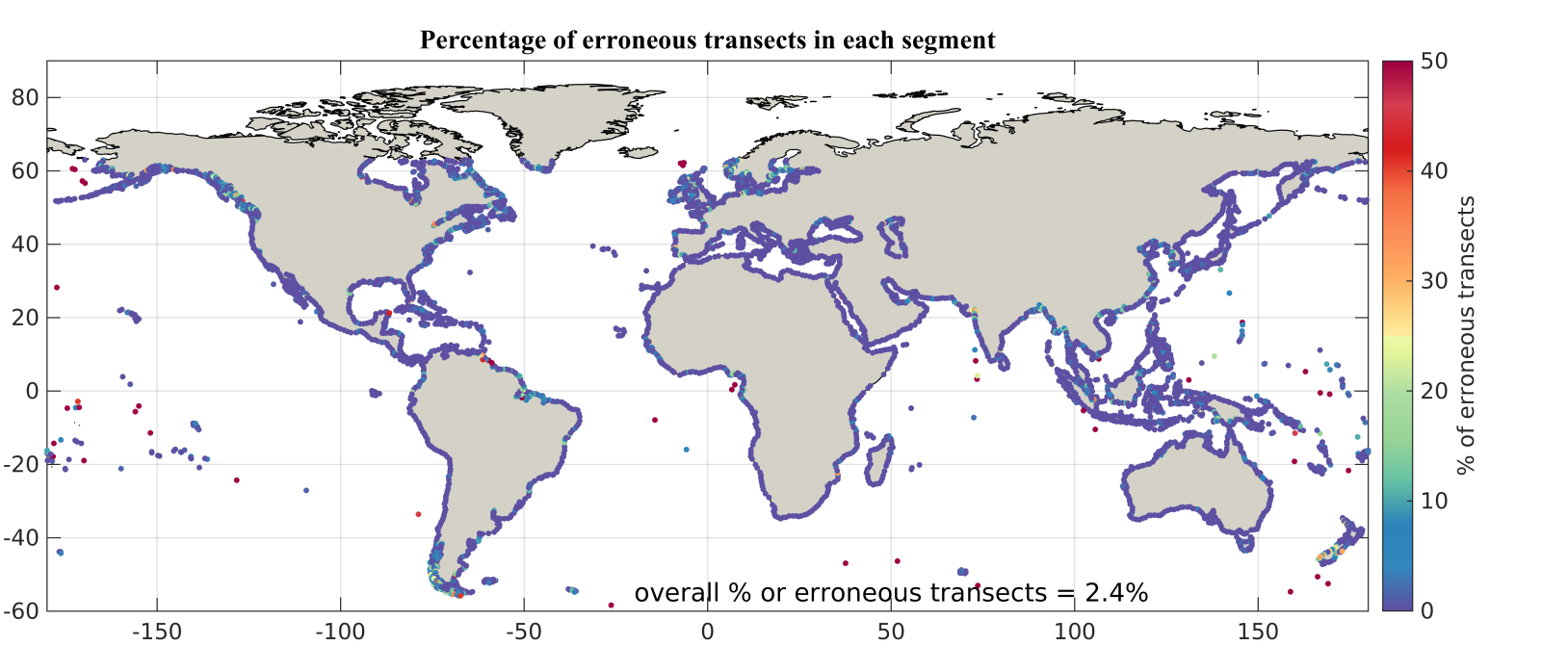
**

Figure 12: Percentage of erroneous transects, i.e. transects not starting on sea or not ending on land, for which a correct estimation of the transition is not possible. This figure was generated with the MATLAB programming language.


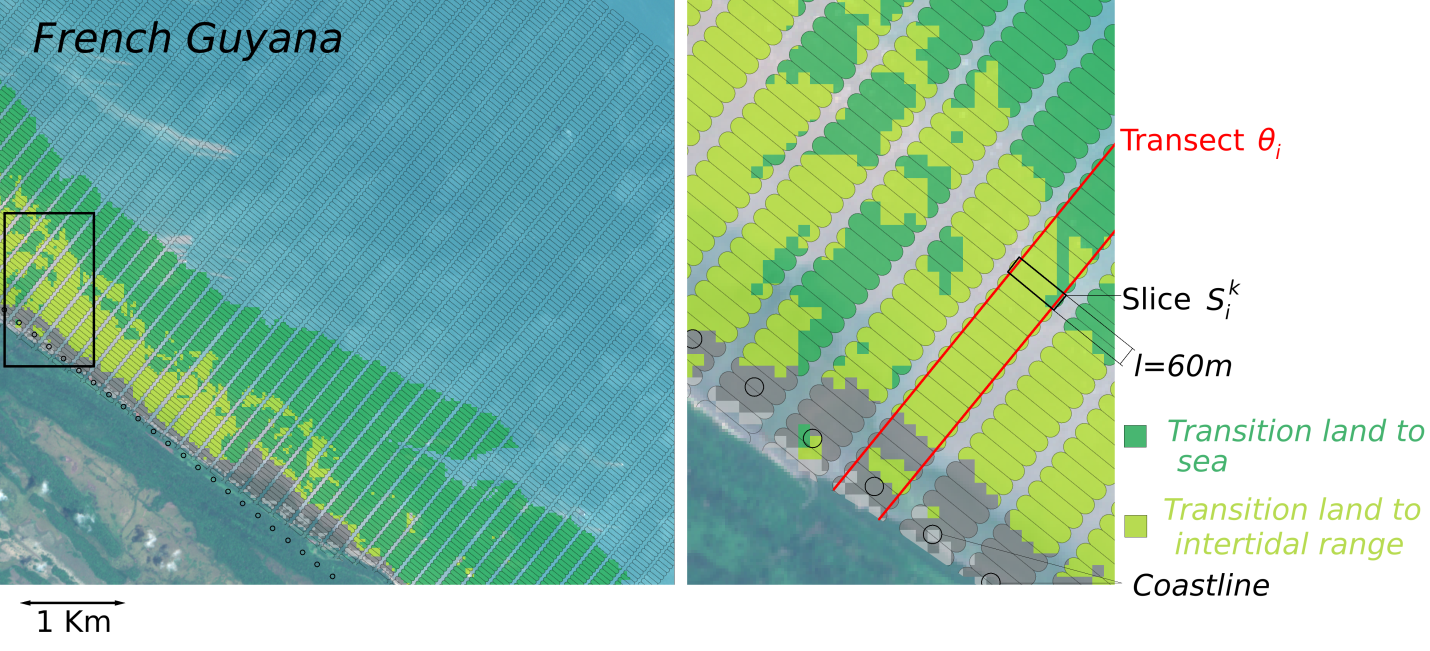


Figure : Coastal analysis algorithm illustrated along the coast of French Guyana. These maps were produced using data from the USGS (http://earthexplorer.usgs.gov/), Copernicus Sentinel data 2016-2017, the Google-Earth-Engine^11^ and the javascript-playground platform.


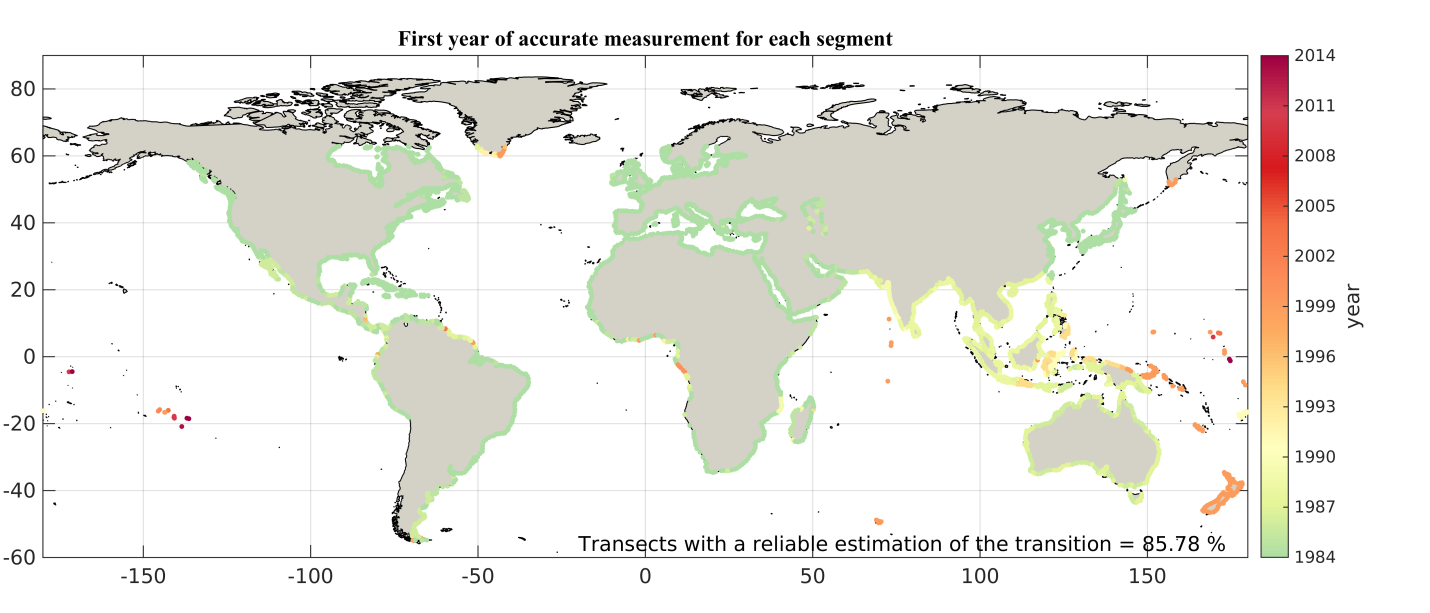


Figure : Transects with a reliable estimation of the transition, and first year of availability of transition data. This figure was generated with the MATLAB programming language.

### References

1. Chu, Z. X., Sun, X. G., Zhai, S. K. & Xu, K. H. Changing pattern of accretion/erosion of the modern Yellow River (Huanghe) subaerial delta, China: Based on remote sensing images. *Mar. Geol.* **227,** 13–30 (2006).

2. Emran, A., Rob, M. A. & Kabir, M. H. Coastline Change and Erosion-Accretion Evolution of the Sandwip Island, Bangladesh. *Int. J. Appl. Geospatial Res.* **8,** 33–44 (2017).

3. Borrero, J. C. Field Data and Satellite Imagery of Tsunami Effects in Banda Aceh. *Science* **308,** 1596–1596 (2005).

4. Anthony, E. J. *et al.* Linking rapid erosion of the Mekong River delta to human activities. *Sci. Rep.* **5,** 14745 (2015).

5. Rosen, T. & Xu, Y. J. Recent decadal growth of the Atchafalaya River Delta complex: Effects of variable riverine sediment input and vegetation succession. *Geomorphology* **194,** 108–120 (2013).

6. Sale, P. F. *et al.* The growing need for sustainable ecological management of marine communities of the Persian Gulf. *Ambio* **40,** 4–17 (2011).

7. Zafar Khan, M. & Akbar, G. in *River Conservation and Management* 69–78 (John Wiley & Sons, Ltd, 2012). doi:10.1002/9781119961819.ch6

8. Turner, I. L. *et al.* A multi-decade dataset of monthly beach profile surveys and inshore wave forcing at Narrabeen, Australia. *Sci. Data* **3,** 160024 (2016).

9. Gorelick, N. Google Earth Engine. *AGU Fall Meet. Abstr.* **15,** 11997 (2012).

10. Watson, C. S. *et al.* Unabated global mean sea-level rise over the satellite altimeter era. *Nat. Clim. Chang.* **5,** 565–568 (2015).

11. Gorelick, N. *et al.* Google Earth Engine: Planetary-scale geospatial analysis for everyone. *Remote Sens. Environ.* **202,** 18–27 (2017).
